# Similar adaptative mechanism but divergent demographic history of four sympatric desert rodents in Eurasian inland

**DOI:** 10.1038/s42003-023-04415-y

**Published:** 2023-01-12

**Authors:** Jilong Cheng, Xingwen Peng, Hong Li, Anderson Feijó, Lin Xia, Georgy I. Shenbrot, Deyan Ge, Zhixin Wen, Dehua Wang, Qisen Yang

**Affiliations:** 1 Key Laboratory of Zoological Systematics and Evolution, Institute of Zoology, Chinese Academy of Sciences, Chaoyang District, Beijing, 100101 China; 2 College of Life Sciences, University of Chinese Academy of Sciences, Shijingshan District, Beijing, 100049 China; 3 Novogene Bioinformatics Institute, Beijing, 100083 China; 4 Mitrani Department of Desert Ecology, Jacob Blaustein Institutes for Desert Research, Ben-Gurion University of the Negev, Midreshet Ben-Gurion, Negev, 84990 Israel; 5 State Key Lab of Integrated management for Pest Insects and Rodents, Institute of Zoology, Chinese Academy of Sciences, Chaoyang District, Beijing, 100101 China

**Keywords:** Comparative genomics, Biodiversity, Evolutionary biology

## Abstract

Phenotypes associated with metabolism and water retention are thought to be key to the adaptation of desert species. However, knowledge on the genetic changes and selective regimes on the similar and divergent ways to desert adaptation in sympatric and phylogenetically close desert organisms remains limited. Here, we generate a chromosome level genome assembly for Northern three-toed jerboa (*Dipus sagitta*) and three other high-quality genome assemblies for Siberian jerboa (*Orientallactaga sibirica*), Midday jird (*Meriones meridianus*), and Desert hamster (*Phodopus roborovskii*). Genomic analyses unveil that desert adaptation of the four species mainly result from similar metabolic pathways, such as arachidonic acid metabolism, thermogenesis, oxidative phosphorylation, insulin related pathway, DNA repair and protein synthesis and degradation. However, the specific evolved genes in the same adaptative molecular pathway often differ in the four species. We also reveal similar niche selection but different demographic histories and sensitivity to climate changes, which may be related to the diversified genomic adaptative features. In addition, our study suggests that nocturnal rodents have evolved some specific adaptative mechanism to desert environments compared to large desert animals. Our genomic resources will provide an important foundation for further research on desert genetic adaptations.

## Abstract (web summary)

Genome assembles for Northern three-toed jerboa, Siberian jerboa, Midday jird and Desert hamster and provides insights into similarities and differences in mechanisms for desert adaptation.

## Introduction

Deserts, known for their harsh environments, cover more than 33% of the world’s land surface^1^. Organisms living in deserts are exposed to aridity and water scarcity, food scarcity, airborne dust, and extreme temperature variation^1,2^. As preserving water and maintaining body temperature are the main challenges to desert species^3^, various complex phenotypes related to metabolism and water retention are generally presumed adaptations of desert species, both in hot deserts and cold-arid deserts^3–5^. Desert rodents are key species and critical components of desert ecosystems. To avoid overheating and to minimize water loss, most desert rodents exhibit nocturnal activity, hiding in burrows during the day, concentrating highly hyperosmotic urine with low volume and utilizing water derived from fat metabolism (metabolic water)^3,4^.

Similar selective pressures in different species can lead to independent evolution of similar phenotypes and/or shared molecular mechanisms to cope with similar evolutionary problems^6^. These species include both distinct phylogenetic lineages with similar phenotypes^7–9^ and monophyletic taxa with a relatively recent common ancestry^10,11^. Similar phenotypes and/or shared molecular mechanisms display clear hierarchical levels, such as nucleotide, gene, pathway, morphology, physiology, biochemistry, and behaviour^6^. Recent studies have demonstrated that convergent and species-specific adaptations in fat metabolism, insulin response, and arachidonic acid (AA) metabolism pathways may be pivotal for mammalian survival in different deserts of the world^2,3,12–15^. However, knowledge on the genetic changes and selective regimes for desert adaptation remains limited in sympatric desert organisms that are phylogenetically close but independently colonize desert niches.

The Eurasian inland possesses the world’s largest mid-latitude arid zone characterized by unique, complex landforms with huge mountains and inland rivers^16,17^. This arid zone was affected long-term by cooler and drier tendencies during the late Cenozoic and the uplift of the Qinghai-Tibetan Plateau^18^. Historically, repeated expansions and contractions of desert–grassland–forest systems and their impact on faunal succession occurred during the alternation of cold and warm periods in the Quaternary climate cycle^19,20^. The unique geological history has shaped the climate difference between the Eurasian inland and low-latitude deserts such as the Sahara Desert. In addition to extreme heat (>50 °C) and drought in summer, the frigid airflow from Siberia results in extreme cold (<−20 °C) and strong winds during winter^17^.

**Fig. 1:**
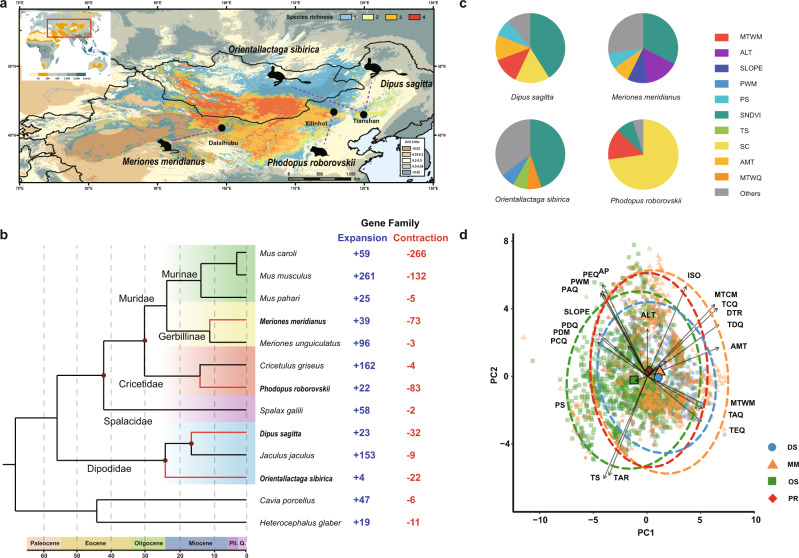
Localities, phylogenetic relationships and convergent selection in ecological factors of the four desert rodents. **a** Sampling locations and distribution ranges of *Dipus sagitta* (DS), *Orientallactaga sibirica* (OS), *Meriones meridianus* (MM), and *Phodopus roborovskii* (PR). The base map shows the arid index. The colour representing the species richness indicates the number of species present in the current region. **b** Phylogenetic tree using the maximum-likelihood algorithm from 5102 single-copy orthologous groups of 13 species. Branch lengths in the tree are scaled to estimate divergence times in Mya. The tree topology is supported by posterior probabilities of 1.0 for all nodes. The numbers of significantly expanded and contracted gene families are labelled following each branch. **c** Contributions of environmental variable to the four species SDM in MAXENT models. The size of the color pie ratio indicates the size of the contribution value. **d** Scatterplot of the first and second principal components of the 21 climatic parameters with 95% confidence eclipses clustered by species. The large colored polygons represent the centroid of each species. The environmental factors included altitude, terrain slope, and 19 climate factors. Detailed information is listed in the Supplementary Note 2.

Jerboas (Dipodidae, Dipodoidea), gerbils (Gerbillinae, Muridae, Muroidea) and hamsters (Cricetinae, Cricetidae, Muroidea) constitute the dominant taxa of sympatric desert rodents in the Eurasian inland^21^. These rodents are all classified as two separate lineages of Dipodoidea and Muroidea in Myomorpha^21^ (Fig. 1a, b). Although they have evolved different morphological types, habitat choices and activity rhythms^21^, they experienced the same geological history background since the late Miocene and independently colonized desert niches (Supplementary Note 1), which provides a well-suited system to study similar and divergent desert adaptation within the context of independent origination.

In this study, we sequenced and generated four high-quality de novo genomes from representatives of four sympatric desert rodents from the Eurasian inland, including Northern three-toed jerboa (*Dipus sagitta*, DS), Siberian jerboa (*Orientallactaga sibirica*, OS), Midday jird (*Meriones meridianus*, MM), and Desert hamster (*Phodopus roborovskii*, PR) (Supplementary Fig. 1), to investigate the molecular mechanism of desert adaptation of the four species. Our results unveil similar adaptative metabolic pathways but divergent evolved genes in AA metabolism, thermogenesis, oxidative phosphorylation, insulin-related pathways, DNA repair and protein synthesis and degradation, which may work together to enable desert rodents to adapt to cold deserts in the Eurasian inland. The niche segregation and demographic histories analyses of the four species revealed similar niche selection but different effective population sizes and sensitivities to climate change, which may be related to the diversified genomic adaptative features.

## Results and discussion

### Species distribution modeling, spatial climate segregation and niche width

To explore the selective regimes of the four species on external environmental factors, we first constructed species distribution modeling (SDM). We obtained a dataset including 22 environmental factors represented by climate, relief, and vegetation variables from 620 localities for DS, 1028 localities for OS, 581 localities for MM and 332 localities for PR, covering most of the species’ distribution ranges (Supplementary Fig. 1). The distribution areas of the four species overlapped widely. The contributions of environmental factors to SDMs showed similarities among the four species. The summer NDVI made important contributions for DS (41.0), OS (44.8), MM (32.5) and PR (8.1), and sand cover contributed significantly to PR (72.7) and DS (16.0) (Fig. 1c). Then, we assessed which set of environmental variables was most closely associated with species distribution via principal component analysis. The bioclimatic space occupied by the four species revealed a large overlap (Fig. 1d), which was consistent with SDM (Supplementary Fig. 1). The distribution of OS was more closely associated with higher-precipitation areas, whereas MM seemed to prefer areas with higher temperatures. Finally, we evaluated the macrohabitat niche breadth of each species. The breadths of environmental space occupation were similar for DS (0.527), MM (0.571), and PR (0.548) and slightly higher for OS (0.622), which suggests that niche selection among the four species is partially overlapping. In total, the four species are mostly similar in the selection of external environmental factors.

### High-quality genomic landscapes of the four desert rodents

**Fig. 2:**
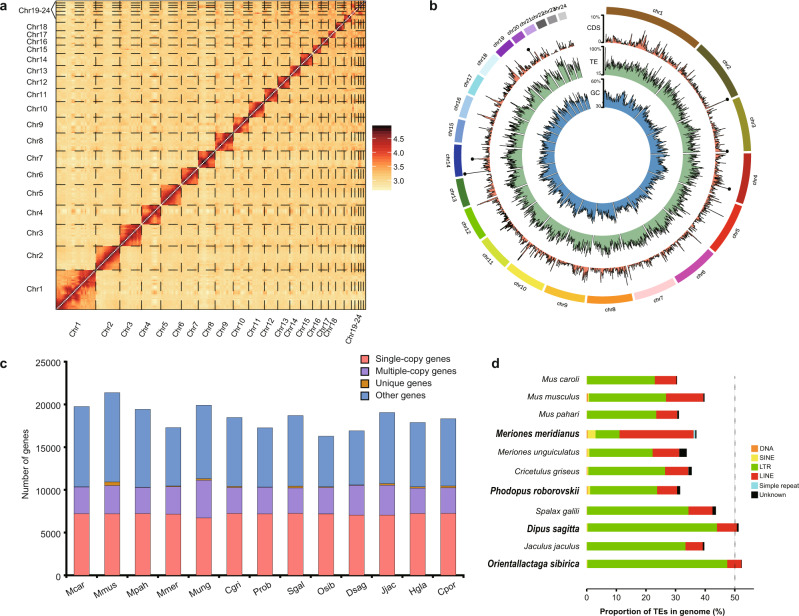
High-quality assembly of *Dipus sagitta* genome and genomic elements of the four sequenced desert rodents. **a** Hi-C heat map of *Dipus sagitta* genome assembly. **b** CIRCOS plot showing the distribution of GC content, transposable elements (TE), and coding sequences (CDS) in the *D. sagitta* genome. **c** Orthologous coding sequences composition inferred for thirteen rodents’ genomes. Mcar *Mus caroli*, Mmus *Mus musculus*, Mpah *Mus Pahari*, Mmer *Meriones meridianus*, Mung *Meriones unguiculatus*, Cgri *Cricetulus griseus*, Prob *Phodopus roborovskii*, Sgal *Spalax galili*, Osib *Orientallactaga sibirica*, Dsag *Dipus sagitta*, Jjac *Jaculus jaculus*, Hgla *Heterocephalus glaber*, Cpor *Cavia porcellus*. **d** Proportion of transposable elements (TEs). The barplots show the proportions of different types of TEs in corresponding species on the phylogenetic tree.

To investigate the genetic mechanism for desert adaptation of the four sympatric desert rodents, we generated four high-quality de novo genomes (Supplementary Fig. 2). The DS was sequenced using a combined strategy and generated 377.67 Gb of data from Illumina reads, 261.01 Gb from PacBio long reads, 299.51 Gb from 10X Genomics reads, and 389.13 Gb from Hi-C reads (Supplementary Table 1). The final genome size was 2.81 Gb with contig N50 of 31.41 Mb and ~472X mean coverage (Table 1, Supplementary Fig. 3, and Supplementary Tables 2, 3). The contigs for DS were further assembled into pseudochromosomes with lengths on the order of full chromosomes and a scaffold N50 size of 147.24 Mb (Fig. 2a, b, Table 1, and Supplementary Fig. 4). The OS, MM and PR were sequenced using the same hybrid strategy and generated 162.58 Gb, 172.22 Gb, and 214.34 Gb Illumina reads and 183.09 Gb, 161.34 Gb, and 186.45 Gb Oxford Nanopore Technologies long reads, respectively (Supplementary Table 1). The final assembly of OS, MM and PR was 2.83 Gb, 2.43 Gb, and 2.16 Gb with contig N50 of 25.87 Mb, 24.08 Mb, and 42.68 Mb, respectively (Table 1, Supplementary Fig. 4, and Supplementary Tables 2, 3).

**Table 1:** Genome assembly statistics of the four desert rodents.

Species	*Dipus sagitta*	*Orientallactaga sibirica*	*Meriones meridianus*	*Phodopus roborovskii*
Assembly length (Gbp)	2.81	2.83	2.43	2.16
Sequence coverage (X)	474.10	123.45	137.26	185.55
Contig N50 (Mbp)	31.41	25.87	24.08	42.68
Scaffold N50 (Mbp)	147.24	—	—	—
GC content (%)	42.16	41.87	42.12	41.38
BUSCO Completeness (%)	93.8	92.9	95.0	95.9

Analyses of the four draft genomes showed that 92.9–95.9% of mammalian BUSCOs were complete, and the GC content was 41.38–42.16% (Table 1 and Supplementary Table 3). Whole-genome annotation was performed via three complementary methods: ab initio prediction, homology-based prediction and RNA-seq based prediction. A total of 23,482, 22,859, 22,533, and 22,314 protein-coding genes were annotated for DS, OS, MM, and PR, respectively (Fig. 2c, Supplementary Fig. 5, Supplementary Table 4). Approximately 98.8–99.1% of genes were functionally annotated for the four species (Supplementary Table 4). Transposable elements (TEs) accounted for 31.38–53.02% of genome assemblies, which predominantly consisted of long-terminal repeats (LTRs), long interspersed nuclear elements (LINEs) and other unknown TEs (Fig. 2d). DS and OS displayed significant LTR expansion of 47.39% and 50.88% in four sequenced genomes, while MM showed an unexpectedly high LINE expansion of 28.99% and sharp LTR contraction to 9.38% (Supplementary Table 5).

### Phylogenetic relationship and evolutionary history

Using 5,102 single-copy orthologous groups, we constructed a high-confidence phylogenetic tree using the maximum-likelihood algorithm, including time calibrations based on fossil records and previous studies (Figs. 1b, 2c)^22^. The phylogenetic tree strongly supported nodes uniting the subfamilies Murinae and Gerbillinae, which together represented the family Muridae (Supplementary Fig. 6). This group was sister to a clade containing cricetids. Spalacidae was recovered as the earliest divergent lineage from Muridae and Cricetidae in the superfamily Muroidea. The split of the most recent common ancestor of Dipodoidea and Muroidea dated to ~56.5 Mya (Fig. 1b, Supplementary Fig. 7). In the Miocene epoch (23 Mya–5.3 Mya), accelerated global geotectonic movement aggravated global climate drying and cooling^23^. Geological disruptions that modified landscapes and offered new habitats favored the early adaptive radiation of extant desert rodents. The ancestors of four sequenced species emerged separately during this period (Supplementary Note 1). Our phylogenetic tree is consistent with previous evolutionary research on rodents^22^ and supports the independent evolution of desert adaptations in Jerboas, Gerbils and Hamsters.

### Expanded and contracted gene families

Comparative genomic analysis revealed 23/32, 4/22, 39/73, and 22/83 gene families exhibiting significant expansion/contraction in the genomes of DS, OS, MM, and PR, respectively (Fig. 1b and Supplementary Fig. 8). Genes belonging to the expanded/contracted families were functionally enriched (Fisher Exact < 0.05) in 127/44, 12/27, 48/81, 55/35 Gene Ontology (GO) terms and 7/15, 7/17, 3/18, and 9/5 KEGG (Kyoto Encyclopedia of Genes and Genomes) pathways (*p* < 0.05) in DS, OS, MM, and PR, respectively (Supplementary Data 1–2). Expanded genes were enriched in pathways including vasopressin-regulated water reabsorption (mmu04962, DS/PR), biosynthesis of unsaturated fatty acids (mmu01040, DS), microtubule-based movement (GO:0007018, DS/PR), kidney development (GO:0001822, DS) and so on, while contracted genes involved in pathways such as drug metabolism-cytochrome P450 (mmu00982, DS), microtubule-based process (GO:0007017, OS/PR), lipid catabolic process (GO:0016042, MM/PR), potassium ion transmembrane transport (GO:0071805, DS) and so on.

### Analysis of selection and convergence

We analyzed the positively selected genes (PSGs) and rapidly evolving genes (REGs) in four desert rodents. The phylogenetic tree used for selection analysis has the same topology as the ML tree. When performing branch and branch-site model tests on one desert species, all other species without three other sequenced species were treated as background. Our analyses yielded 1972/1993/1547/1383 PSGs and 694/751/664/583 REGs for DS/OS/MM/PR (Supplementary Data 3–10). Among these genes, 55 PSGs and five REGs were shared by all four desert rodents (Supplementary Fig. 9, Supplementary Table 6).

The convergent/parallel evolution can be evidenced by identifying the same or similar amino acid substitutions of protein-coding sequences, transcriptional regulation of gene expression and similarly enriched metabolic pathways^10,24,25^. To test whether the three sympatric representative desert rodent taxa have convergent genetic changes to cope with similar challenges, we used the JTT-Fgene model^26^ and the PCOC method^27^ to identify genes that are under convergent evolution. PR represented the hamster group, the ancestral clades of MM and Mung represented the gerbil group, and the ancestral clades of DS, OS and Jjac represented the jerboa group (Fig. 1b). We first compared the observed number of convergent amino acid substitutions examined with neutral expectations between the three representative clades in the JTT-Fgene model. A total of 575 genes were identified under the JTT-Fgene model (*p*-value < 0.05, FDR < 0.05, Passion test, Supplementary Table 7). Three genes (*ABTB1*, *SUSD4*, and *SYNE3*) were identified to be under convergent evolution among the three taxa. The PCOC method considered shifts in amino acid preference instead of convergent substitutions^28^. We used the PCOC method to verify the convergent gene set obtained by JTT-Fgene model and detected 442 genes undergoing convergent evolution by both methods (posterior probability > 0.95, Supplementary Table 7).

### Adaptations in the Arachidonic acid metabolism pathway

**Fig. 3:**
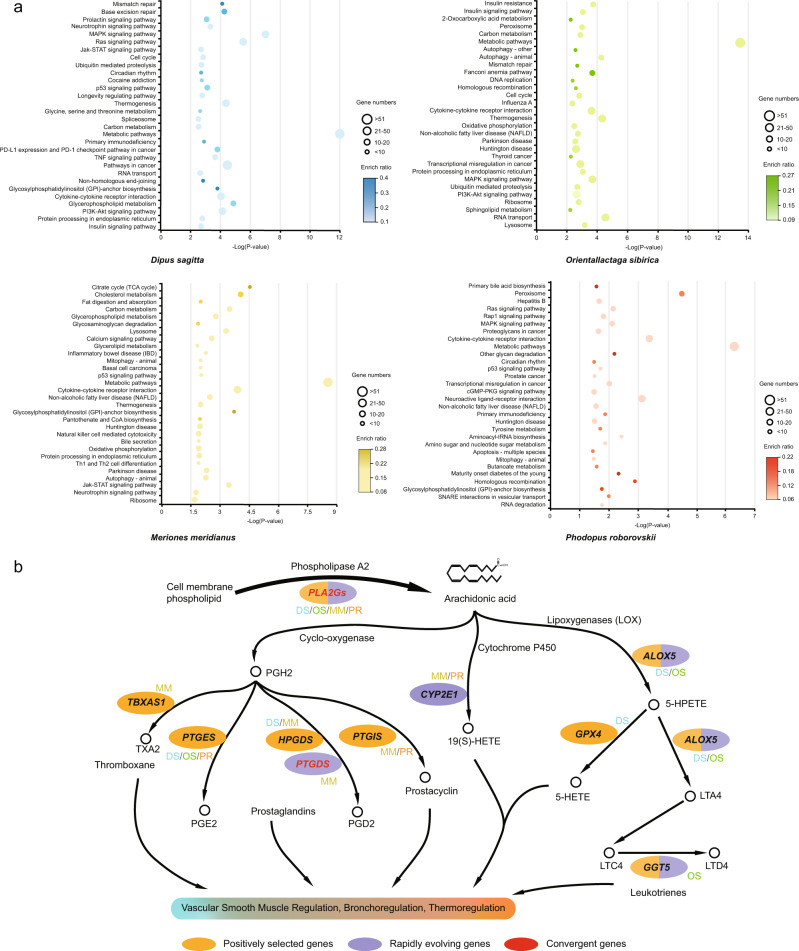
The enriched KEGG pathways and diagram of arachidonic acid metabolism pathway evolution in the four desert rodents, *Dipus sagitta* (DS), *Orientallactaga sibirica* (OS), *Meriones meridianus* (MM), and *Phodopus roborovskii* (PR). **a** The enriched KEGG pathways of combined positively selected genes (PSGs) and rapidly evolving genes (REGs) in the four desert rodents. The x axis shows the -Log (*P*-value) of PSGs and REGs in each pathway. The size of the dot indicates the number of genes. The depth of dot color indicates the enrich ratio. **b** Alterations in the arachidonic acid metabolism pathway of the four desert rodents. Genes in yellow background are PSGs, genes in violet background are REGs, and red genes are under convergent evolution. The symbol next to the gene indicates the species in which the gene has evolved.

The AA metabolism pathway (AAMP) has been shown to be the primary adaptation pathway in almost all desert species^12–15^. We first investigated the selective signatures in AAMP across the four species and whether the evolved genes were in line with previous studies in some large desert-adapted animals. We examined 86 genes that belong to AAMP (KEGG pathway: mmu00590) to seek genetic signatures of adaptive evolution. Although the KEGG functional enrichment of AAMP could be observed for PSGs, REGs (except in DS), and convergent evolution genes, they were not significant (*p* > 0.01) (Supplementary Data 11). We screened the PSGs and REGs and found that the adaptive evolution of this pathway mainly focused on the conversion of lecithin to AA and the production of AA’s cyclo-oxygenase and lipoxygenase products (Fig. 3). DS showed positive selection in seven loci (*PLA2G2F*, *PLA2G4C*, *PLA2G6*, *GPX4*, *ALOX5*, *PTGES2*, *HPGDS*), OS showed positive selection in five loci (*PLA2G1B*, *PLA2G3*, *GGT5*, *ALOX5*, *PTGES*) and rapid evolution in two (*GGT5*, *ALOX5*), MM showed positive selection in four loci (*PLA2G1B*, *TBXAS1*, *HPGDS*, *PTGIS*) and rapid evolution in two (*CYP2E1*, *PTGDS*), and PR showed positive selection in two loci (*PTGES*, *PTGIS*) and rapid evolution in two (*PLA2G4F*, *CYP2E1*) (Fig. 3b).

The phospholipase A2 (*PLA2Gs*) is involved in the hydrolyzation of cell membrane phospholipids, thereby releasing the polyunsaturated fatty acid (AA), which is one of the important sources of AA in organisms^29^. *PLA2Gs* showed positively and rapidly involved genetic signatures in all four species. In particular, *PLA2G2F* has also undergone convergent evolution between hamsters and jerboas (Fig. 3b; Supplementary Table 7). Similar selection signatures of *PLA2Gs* were also identified in desert adaptation animals such as dromedary (*PLA2G2F*)^2^, Tarim red deer (*PA2GF* and *PA2GC*)^14^, and Saudi Arabian indigenous chicken (*PLA2G12B*)^30^. After the formation of AA, it could be further metabolized into different eicosanoids, such as hydroxyeicosatetraenoic acid, thromboxanes and prostaglandins, and leukotrienes^31^. Genes of the cytochrome P450 (CYP) family can help to transform AA into 19(S)-HETE, which is a potent vasodilator of the renal preglomerular vessels that stimulates water reabsorption^32^. Although 19(S)-HETE is thought to play an important role in the survival of desert animals such as camels^12^, sheep^15^, and Tarim red deer^14^, only *CYP2E1* in the cytochrome P450 pathway showed rapidly evolving selection in MM and PR, and the cytochrome P450 gene family showed contraction in all four desert rodents.

Thromboxanes and prostaglandins can influence endothelial and vascular smooth muscle cell function and then indirectly regulate blood pressure, water retention and reabsorption, and immediate hypersensitivity reactions^33^. Therefore, it is thought that selective signals related to vascular smooth muscle regulation or bronchoregulation may be associated with desert adaptation, such as asthma caused by airborne dust^2,14^. Cyclooxygenase metabolizes AA to prostaglandin H2 (PGH2), which is further converted into biologically active prostaglandins^33^. We detected selection signatures for some genes (*TBXAS1*, *PTGES*, *PTGES2*, *HPGDS* and *PTGDS*) involved in the bioactivation of PGH2. For example, *PTGES/PTGES2* convert PGH2 to prostaglandin E2 (PGE2), which plays a key role in thermoregulation, inflammation response, and bronchodilatation^34,35^, as well as increases renal blood flow and provokes dieresis, natriuresis, and kaliuresis^36^. Although prostaglandins have a similar role in vascular smooth muscle tone regulation as angiotensin and 19(S)-HETE, adaptative signatures of genes in the cyclooxygenase pathway has rarely been mentioned in previous studies of desert animals^12,13,15^. In addition to biologically active prostaglandins, leukotrienes, which are lipid mediators of inflammation and chemotaxis, are also potent bronchoconstrictors that play an important role in immediate hypersensitivity reactions^37^. We detected selection signatures in lipoxygenase pathways (*ALOX5*, *GGT5*, *GPX4*) that produce leukotrienes, which are different from selective signatures such as *TRAF2* in Tarim red deer^14^ or functional enrichment such as lung development (GO:0030324) in camels^2^.

The entire AAMP undergoing convergent evolution in desert-adapted mammals and birds has become a consensus^3,12,13,15^. However, in the selection of downstream AAMP, the specific genes involved differed among species and in the mechanisms by which they putatively affected adaptive phenotypes. For example, desert rodents seem to show more selective pressure on the prostaglandins and leukotrienes pathway (Fig. 3b) rather than selecting on cytochrome P450 in desert-adapted large herbivores or chickens^12,14,15,30^. Therefore, we hypothesized that regulation of biologically active prostaglandins might be another adaptative way to desert environments in desert organisms. There are also differences in selection preferences among the four sequenced rodents, such as the lipoxygenase downstream pathways showing selection signatures and functional enrichment “inflammatory response” (GO:0006954) only in jerboas (Supplementary Fig. 10, Supplementary Data 12). This may suggest that similar adaptive phenotypes in different desert organisms may be achieved through a combination of convergent and idiosyncratic adaptations.

### Adaptations in thermostasis and energy homeostasis

**Fig. 4:**
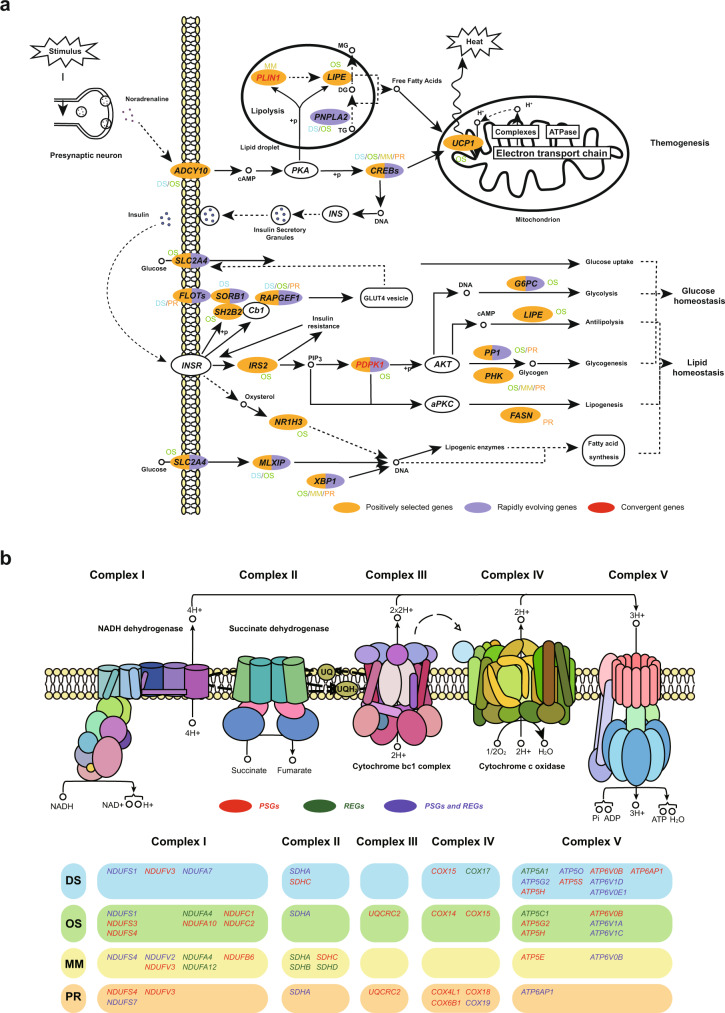
Schematic mechanisms of thermostasis and energy homeostasis pathways in the four desert rodents. **a** Alterations in the thermogenesis pathway, insulin related pathway, and non-alcoholic fatty liver disease pathway of the four desert rodents. Genes in yellow background are PSGs, genes in violet background are REGs, and red genes are under convergent evolution. The symbol next to the gene indicates the species in which the gene has evolved. DS, *Dipus sagitta*, OS, *Orientallactaga sibirica*, MM, *Meriones meridianus*, PR, *Phodopus roborovskii*. **b** Evolved genes in the oxidative phosphorylation of the four desert rodents. Genes in red are PSGs, genes in green are REGs, and violet genes are under both positive selection and rapidly evolving. Different colored bars represent different species. The five sections of the same colored bar correspond to the five complexes. The underlying graph of the mitochondrial electron transport chain is from the KEGG website (https://www.genome.jp/pathway/mmu00190).

Most desert rodents are nocturnally activity and hide in burrows during the day to avoid overheating. Although the daytime temperatures were extremely high in the four desert rodent habitats, the Eurasian inland where they were distributed was a cold desert with extreme diurnal ambient temperature variation. Adaptive thermogenesis is essential for them to ensure normal cellular and physiological function under conditions of environmental stresses^38^. Among the four species, we found similarly significant enrichment of functional pathways (*p* < 0.05) associated with thermic and energy processes, including 65 PSGs/REGs in the thermogenesis pathway (mmu04714), 34 PSGs/REGs in the oxidative phosphorylation pathway (mmu00190), 12 PSGs/REGs in the citrate cycle (TCA cycle) pathway (mmu00020) (Figs. 3a, 4a, Supplementary Data 11–13). In the presence of changes in ambient temperature, alterations in food intake, and/or sympathetic nervous stimulation, brown adipose tissue (BAT) activity increases resulting in heat generation (Fig. 4a). Upon activation by long-chain fatty acids, *UCP1* transports protons through the inner mitochondrial membrane and uncouples mitochondrial substrate oxidation from ATP production thereby releasing the energy of fatty acid oxidation as heat^39^. Therefore, the oxidative phosphorylation pathway produces ATP to maintain physiological metabolism while also producing protons to maintain the proton gradient on both sides of the mitochondrial inner membrane (Fig. 4b). It is noteworthy that DS and OS showed more selective genes than the other two desert rodents (Supplementary Data 11), which may be because jerboas undergo hibernation during winter. As hibernating animals require intense thermogenesis to arouse from the deep torpor when they are hypothermic (as low as 4–6 °C), both shivering and nonshivering mechanisms need to be invoked rapidly (within a few hours) to raise the body temperature during arousal^40^. In addition to the four species, sweep signatures associated with cold-induced thermogenesis was also detected in other desert rodents^10^. In contrast, none of the small desert rodents^10,13^ showed enrichment on melanogenesis pathway compared with desert-adapted herbivores or chickens^14,30,41^.

Moreover, the uptake and homeostasis of energy is important for desert animals living in food-deprived deserts. The scarcity of water and resources drives metabolism to rely on endogenous nutrients (carbohydrates, lipids, and proteins). The genome-wide features of the four species indicate that both “proteins-for-water”^42^ and “lipids-for-torpor”^43^ metabolic regulation contribute to desert adaptation. The transition to protein catabolism during early resource restriction may be an important water source for desert species^10^. Lipid acquisition and storage are important to extreme environmental-induced torpor because they enable desert species to maintain a low metabolic state for a long time^10,43^. For example, *ACOT1*, which catalyzes the hydrolysis of long-chain fatty acyl-CoAs into free fatty acids and coenzyme A^44^, was identified by KEGG pathways in fatty acid elongation (mmu00062, DS) and biosynthesis of unsaturated fatty acids (mmu01040, DS) (Supplementary Data 1), while functional enrichments of contracted gene families were identified in lipid-related GO categories (lipid catabolic process, GO:0016042, MM/PR; medium-chain fatty acid metabolic process, GO:0051791, MM) and protein-related pathways (cysteine and methionine metabolism, mmu00270, DS/MM; tryptophan metabolism, mmu00380, DS/MM; proteolysis, GO:0006508, PR) (Supplementary Data 2). The positively selected and rapidly evolving functional enrichments associated with endogenous nutrient metabolism of the four desert rodents included insulin signaling pathway (INSP) (mmu04910, DS/OS), non-alcoholic fatty liver disease (NAFLD) (mmu04932, all four species), lipid metabolism (mmu00561, DS/OS/MM; mmu00564, DS/OS/MM; mmu04979, OS/MM/PR; mmu00600, OS/PR; GO:0006629, all four species), lipid and cholesterol homeostasis (GO:0042632, OS/MM/PR; GO:0055088, OS), amino sugar and nucleotide sugar metabolism (mmu00520, OS/PR), proteolysis (GO:0006508, all four species), and so on. The convergent feature genes also demonstrated an enrichment of KEGG pathways and GO terms associated with metabolism or homeostasis of proteins or amino acids (mmu00330, mmu00270, GO:0006547, GO:0006548), lipids (mmu00600, GO:0090336), and carbohydrates (mmu00520, mmu00051, GO:0042593, GO:0045721) (Supplementary Data 13).

Many genes with selection signals or convergent features were functionally enriched in the INSP and NAFLD pathways in the four desert rodents, although the evolved genes in each species were not fully identical (Figs. 3a, 4a). The insulin-related pathway has been considered genetic bases for previously identified adaptative phenotypes, including energy storage, low energy expenditure, adaptive tolerance to starvation and dehydration^2,3,10,45^. Homeostasis of carbohydrates and lipids via INSP is achieved by regulating downstream pathways such as glucose uptake, glycolysis, glycogenesis, antilipolysis, lipogenesis and fatty acid synthesis. For example, *G6PC* (OS), hydrolyzing glucose-6-phosphate to glucose in the endoplasmic reticulum (ER), is the key enzyme in the homeostatic regulation of blood glucose levels^46^; *LIPE* (OS), with broad substrate specificity, catalyzes the hydrolysis of several lipids^47^; *FASN* (PR) catalyzes the de novo biosynthesis of long-chain saturated fatty acids in the presence of NADPH^48^. NAFLD, for which selection signals have been observed only in desert rodents, also plays a role in lipid homeostasis (Supplementary Data 11)^10^. The induction of insulin resistance activates key enzymes of lipogenesis and increases the synthesis of free fatty acids in liver leading to excess lipid accumulation^49^. For instance, *NRI3H* (OS) induces phospholipid remodeling in hepatocytes^50^; *MLXIP* and *MLXIPL* (DS and OS) play roles in the transcriptional regulation of glycolytic target and glucose-responsive genes (Supplementary Data 3–10)^51^.

### Adaptations in stress response

Although desert rodents are mostly nocturnal, they are still not immune to the stress of heat and aridity from the regional environment. Thermal stress suppresses the transcription and translation machinery, increases DNA breaks and protein oxidation, alters cell morphology and cell adhesion, causes cell cycle arrest, and eventually leads to apoptosis and cell death^52^. Hyperosmotic stress caused by water shortage and water loss indirectly accelerates the rapid accumulation of cellular damage and exacerbates heat killing^53^. Repair of the genetic expression process has always been an important mechanism for animals to adapt to extreme environments^2,11,13,54^. Our results indicated that many genes with the strongest selection signatures were enriched in the GO terms and KEGG pathways related to various signaling pathways and different stages of genetic expression processes (Supplementary Data 11–13).

In the DNA replication and repair stage, many PSGs/REGs were enriched in multiple GO categories related to different types of DNA repair, as well as in KEGG pathways such as base excision repair (mmu03410) and homologous recombination (mmu03440) (Fig. 3a, Supplementary Fig. 10, Supplementary Data 14). Among these PSGs/REGs, four genes (*LIG3*, *MUTYH*, *POLL*, *POLM*) were also under convergent evolution and separately involved in base excision repair (*LIG3*, *MUTYH*), DNA double-strand break repair (*POLM*) or both (*POLL*) (Supplementary Data 14)^55,56^. Although DNA repair-related pathways are enriched in all four species and partly overlap among different species, genes under selection pressure in these pathways are not the same in the four species. The number of PSGs/REGs was significantly higher in jerboas, which also shared more PSGs/REGs.

In the transcription stage, a large number of pathways related to transcription and splicing were enriched in gene family evolution, selective evolution and convergent evolution, such as regulation of transcription, DNA-templated (GO:0006355 in four species), regulation of transcription by RNA polymerase II (GO:0006357 in DS, OS and MM), spliceosome (mmu03040 in four species), mRNA processing (GO:0006397 in DS and OS), RNA splicing (GO:0008380 in DS and OS), mRNA transport (GO:0051028 in OS and PR) (Supplementary Data 11–13). Similarly, all analyses indicated that traits associated with the synthesis and degradation of proteins have evolved under the influence of natural selection. The extended or contracted gene families were enriched in various GO categories and KEGG pathways, such as proteasome (mmu03050 in PR), phagosome (mmu04145 in DS, OS, and PR), apoptosis (mmu04210 in OS), and protein processing in endoplasmic reticulum (mmu04141 in MM) (Supplementary Data 1–2). The PSGs/REGs and convergent genes also demonstrated an enrichment of terms and pathways associated with this process, such as protein processing in endoplasmic reticulum (mmu04141 in DS, OS and MM), lysosome (mmu04142 in OS, MM and PR), ubiquitin mediated proteolysis (mmu04120 in DS and OS), ribosome (mmu03010 in OS and MM), proteolysis (GO:0006508 in four species), protein ubiquitination (GO:0016567 in DS, OS and MM) (Supplementary Data 11–13).

In the regulation of genetic expression processes, various signaling pathways are commonly linked by some of the same node genes and cascade reactions, which jointly regulate individuals’ responses to extreme environmental stress. For example, *CREB* is a phosphorylation-dependent transcription factor that stimulates transcription upon binding to the DNA cAMP response element (CRE)^57^. It is enriched in at least 20 pathways in each species, most of which are signaling pathways such as the insulin signaling pathway, PI3K-Akt signaling pathway, and AMPK signaling pathway (Supplementary Data 11–13). These signaling pathways did not directly affect some adaptive phenotypes, but indirectly affected several key traits of survival in hot arid environments by regulating downstream genes^10,14,15,41^, including energy metabolism, vascular smooth muscle tone regulation, repair of genetic expression processes, thermotolerance, or thermogenesis.

### Similar results but divergent adaptative routes

Although all kinds of desert animals are adapted to desert environments, it seems that desert rodents have chosen different evolutionary routes in phenotypes and genotypes^2,13–15^. Taking heat regulation as an example, some large desert animals have adaptive evolution in storing heat without increasing body temperature^3^, the melanogenesis pathway^14,28,41^, photoreception and visual protection^2,14^ and other characteristics, while desert rodents have adaptative selection in thermogenesis (Supplementary Data 11–13). Regarding the water retention and utilization, previous studies and our results have found that more traits associated with the synthesis and degradation of proteins in desert rodents have evolved under the influence of natural selection^10,13^, while some large desert animals have relatively more evolved genes and functionally enriched pathways related to water balance and reabsorption, as well as osmoregulation^2,14,15,41^.

The three desert rodent taxa from the Eurasian inland show a potential framework of convergent or parallel evolution as well as similar selection on external environmental factors. However, our results suggest that the same adaptative molecular pathways could be achieved through several genomic routes instead of identical replacements of single amino acids within the encoded product of a protein-coding gene^58^. In macroecology, species with overlapping niches may come into competition with each other when they exploit the same limiting resources^59^, while niche segregation facilitates coexistence^60^. Here, we propose a hypothesis: if metabolic pathways are treated as kinds of resources (genomic niches), whether ecologically similar sympatric species are subjected to different evolutionary selection in the same metabolic pathway can be interpreted as genomic niche separation promoting their coexistence.

### Demographic history

**Fig. 5:**
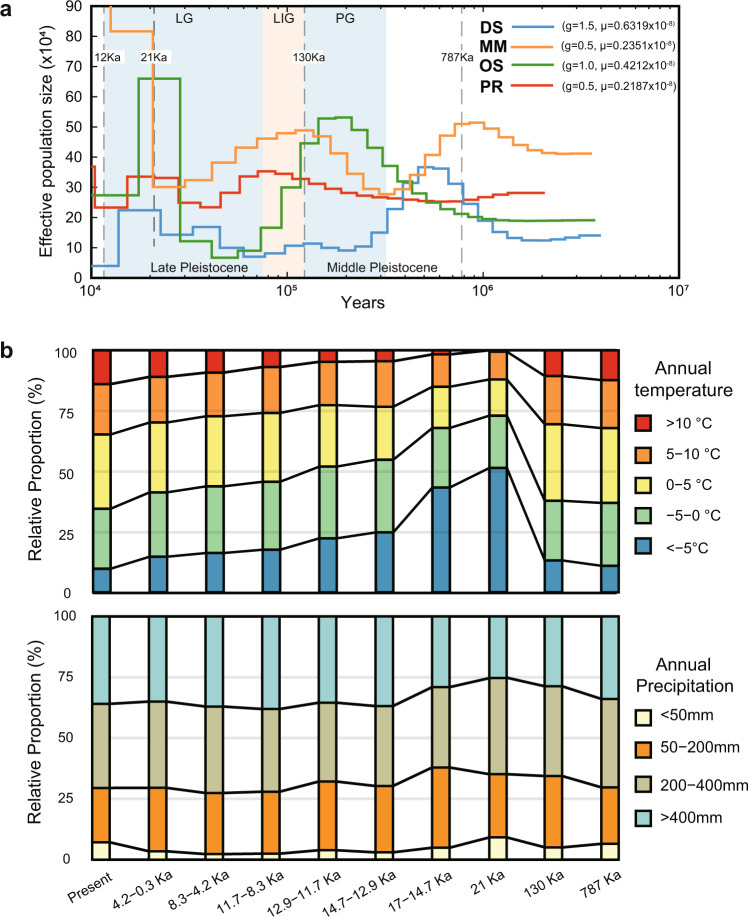
Demographic history of the four desert rodents during historical climate changes. **a** Effective population sizes of the four desert rodents reconstructed using the PSMC model (g, generation time, years; μ, neutral mutation rate per generation). The period of the Penultimate Glaciation (PG, 330–130 ka), and the Last Glaciation (LG, 75–10 ka) are shaded in light blue, and the period of the Last Interglaciation (LIG, 130–800 ka) is shaded in light orange. **b** The relative temporal changes in annual precipitation and annual mean temperature from 787 Ka to present within a defined region (Lat: 33 N, 55 N; Long: 70E, 130E) including the distributions of the four desert species. The annual mean temperature and annual precipitation layers during the Quaternary were downloaded from PaleoClim database.

Quaternary climatic cycles have a profound influence on the formation and current distribution of species^61^. During the Middle Pleistocene (787–130 ka), the climate of the Eurasian inland gradually tended to be dry-cool, even though there were climate cycles. In the late Pleistocene (130–10 ka), the climate reached maximum dry cooling at 21 ka and then began to change rapidly to warm and humid characteristics (Fig. 5). To determine the relationships between the population demographic size and environmental dynamics over time, we estimated the effective population size (Ne) and climate temporal shifts for the four species over approximately the last one million years using the pairwise sequential Markovian coalescent (PSMC) method^62^. Our PSMC results show that the DS’s expansion began at ~1.0 Mya and presents an overall declining trend (Fig. 5). The OS’s effective population size showed drastic fluctuation with two expansions and two declines. The population expansion of OS significantly coincided with the expanded dry-cooling climate (Fig. 5). Moreover, during the period from 21 Ka to 12 Ka, there were significant decreases in cold temperatures (<−5 °C) and arid regions (< 400 mm) and sharp population reductions. This revealed that OS is more sensitive to warmer temperatures and wetter environments than the other species. MM almost reached the first expansion peak at ~1.1 Mya, followed by another two expansions. The PR’s expansion apparently began at ~500 Ka, followed by another two expansions. Although the macrohabitat choice and genomic features of the four species showed similar evolved selection in desert adaptation, they exhibited different dynamic patterns and relationships with temporal climate changes. We infer that the diversified genomic adaptative features lead to the different demographic histories. In total, all four species experienced repeated fluctuations in effective population size during the Quaternary climatic cycles, which may be related to the historically repeated expansions and contractions of desert–grassland–forest systems in the Eurasian inland. DS, OS, and PR appear to have declined in population size since the Last Glacial Maximum. In contrast, MM had a much larger overall effective population size than other species, which may be due to a broader gene pool of variation for selection^10^. This could be beneficial for MM to adapt to more diverse habitat types in the desert environment, which is consistent with our observations in the field. The population sizes of the two jerboas appeared to be more sensitive and vulnerable to fluctuations in environmental factors than MM.

## Conclusion

In this study, we generated high-quality genomes of two jerboas, a jird and a hamster, representing the dominant taxa of sympatric desert rodents in the Eurasian inland. Niche occupation analysis showed similar selection on external environmental factors of the four species. Genomic analyses revealed that the evolution of desert adaptation in these four species was similar in metabolic pathways, such as arachidonic acid metabolism, thermogenesis, oxidative phosphorylation, insulin-related pathways, DNA repair and protein synthesis and degradation. In addition, our study indicated that there are differences in adaptation to cold and hot deserts, especially for nocturnal rodents. We also found that the four desert rodents exhibited different demographic histories and relationships with historical temporal climate changes. The two jerboas were more sensitive to historical environmental changes, while the midday jird benefitted more from these changes. Our study adds genomic resources to further understand the genetic mechanisms of adaptations in desert animals and lays the foundation for further research on their potential application in the protection of genetic resources in view of ongoing global climate change.

## Materials and methods

### Species distribution modeling, spatial climate segregation and niche width

We first built occurrence points data sets for the four species used in the following analysis. In total, 620 points were used for DS, 1028 points for OS, 581 points for MM and 332 points for PR. Environmental data for species distribution modelling (SDM) were used in the form of 30 arc-second grids (approximately 1 km resolution) and were represented by climate (BioClim 1–19), relief (altitude and slope) and vegetation variables (Normalized Difference Vegetation Index, NDVI). The SDMs were built using MAXENT 3.4.0 software^63^. All map operations were performed using ArcMap 10.8.1 software. We compared the contributions of environmental factors to SDMs among the four species. Then we evaluated which set of environmental variables were most closely associated with species distribution via principal component analysis. Last, we calculated the niche breadth of the four species. To eliminate the influence of factor intercorrelation, original environmental variables were normalized and then ordinated by the principal components analysis (PCA) using the Spatial Analyst module of ArcGIS. The niche breadth was estimated in the space of the first two principal components of environmental variables using kernel smoothing of the densities of species occurrence points^64,65^. All analyses of SDM, spatial climate segregation and niche width were described in detail in Supplementary Note 2.

### Sampling and genome sequencing

We obtained muscle samples from one Northern three-toed jerboa (*Dipus sagitta*) and one Siberian jerboa (*Orientallactaga sibirica)* from Tianshan, Inner Mongolia, China, one Midday jird (*Meriones meridianus*) from Dalaihubu, Inner Mongolia, China, one Desert hamster (*Phodopus roborovskii*) from Xilinhot, Inner Mongolia, China. All collected individuals were female. All procedures were conducted in accordance with Animal Research Protocol IOZ-2006 approved by the Animal Care Committee of the Institute of Zoology, Chinese Academy of Sciences (IOZCAS). DNA was extracted with a Qiagen DNA purification kit (Qiagen, Valencia, CA, USA).

For all four species, we constructed 350 bp short-insert libraries for Illumina sequencing on an Illumina NovaSeq 6000 platform. The following strategies were used to filter the Illumina raw data: (a) reads with ≥10% unidentified nucleotides (N); (b) reads with adapters; and (c) reads with low-quality bases (≤5) accounting for more than 50% of the total length.

The DS genome were sequenced with a hybrid strategy. For PacBio library construction, the genome DNA was sheared to ~20 kb. Single-molecule real-time (SMRT) sequencing was conducted on a PacBio Sequel II sequencing platform. For the 10X Genomics, long DNA insert library was processed by applying the 10X Genomic Chromium system. Long DNA fragments were reduced to 600 bp sizes after barcoding. Finally, the 10X Genomic libraries were quantified and sequenced on the Illumina NovaSeq 6000 platform. Hi-C experiments were used for chromosome assembly of DS. Hi-C Libraries were prepared using the *Mbo* I restriction enzyme. DNA fragments were purified and then fragmented to sizes of 300–500 bp. Finally, the Hi-C libraries were quantified and sequenced on the Illumina NovaSeq 6000 platform.

For the OS, MM and PR, approximately 15 µg genomic DNA of each species was processed according to the Ligation Sequencing Kit 1D (SQK-LSK109) protocol to generate Oxford Nanopore long reads. Oxford Nanopore sequencing adapters were ligated using the NEBNext Quick Ligation Module (E6056) (New England Biolabs). The final library was sequenced using a PromethION DNA sequencer (Oxford Nanopore, Oxford, UK). Guppy software (https://community.nanopore-tech.com/posts/guppy-v4-0-11-release) was used to conduct base calling on the raw signal data. The obtained data were then filtered to remove reads containing adapter sequences.

### RNA sequencing

The same individuals used for genomic sequencing were used for transcriptome sequencing, providing necessary gene expression data for genome sequence annotation. Six tissues from four individuals, including heart, kidney, liver, lung, muscle, and spleen tissues, were collected for subsequent transcriptome sequencing. Sequencing libraries were generated using the NEBNext® Ultra™ RNA Library Prep Kit for Illumina® (NEB, USA) following the manufacturer’s recommendations, and index codes were added to attribute sequences for each sample. After end-repair, adapter ligation, and PCR amplification, each paired-end cDNA library was sequenced with a read length of 150 bp using the Illumina NovaSeq 6000 sequencing platform.

For the DS, additional long-read RNA sequencing (Iso-seq) was conducted. Total RNA was pooled from the same six tissues (heart, kidney, liver, lung, muscle, and spleen). A total of 5 µg RNA per sample was used as the input material for RNA sample preparation. First-strand cDNA synthesis was conducted with the Clontech SMARTer PCR cDNA Synthesis Kit. CDS Primer IIA was first annealed to the polyA tail of the transcripts, followed by first-strand synthesis with SMARTScribeTM reverse transcriptase. The first-strand product was diluted with elution buffer (EB) to an appropriate volume and subsequently used for large-scale PCR. Once double-stranded cDNA was prepared, the PacBio Template Prep Kit was used to generate SMRTbellTM libraries. The SMRTbell templates were then sequenced on the PacBio Sequel II platform.

### De novo assembly of the four genomes

Genome size were predicted according to k-mer genome survey in Jellyfish^66^. The genome sizes of the four species (DS, OS, MM, and PR) were estimated to be 2.91, 3.19, 2.61, and 2.38 Gb, respectively (Supplementary Fig. 11). For the DS genome, we used wtdbg2^67^ to assemble the PacBio long sequencing data. As the high random sequencing errors of PacBio raw reads, Quiver^68^ was used to correct the original assembly, followed by further correction with Illumina short clean reads using Pilon^69^. Then, the primary contigs from the wtdbg2 assembly and 10X Genomic clean reads were used as inputs for fragScaff software (https://sourceforge.net/projects/fragscaff) with the parameters ‘-maxCore 200 -m 3000 -q 30 -C 5’. The scaffolding procedure was as follows: 1) Linked-reads generated using the 10X Genomics library were aligned to the PacBio assembly result to obtain the super scaffold using BWA v0.7.8^70^. 2) After scaffolding with 10X Genomic reads, the resulting scaffolds were used as the input to generate a chromosomal-level genome assembly containing Hi-C clean reads. All Hi-C clean data were aligned to the scaffolding assembly using BWA v0.7.8^70^ software with the default parameters. HiCUP v0.5.9^71^ software was used to evaluate the valid Hi-C data based on uniquely mapped read pairs. Only valid read pairs were used for draft genome re-correction and chromosome-level genome assembly. According to cross-linked long-distance physical interactions, LACHESIS^72^ was used to cluster scaffolds into groups with the following parameters: RE_SITE_SEQ = GATC; CLUSTER_N = 23; and CLUSTER_MIN_RE_SITES = 50. To evaluate the quality of the chromosomal-level genome assembly, a genome-wide Hi-C heatmap was generated using ggplot2 in the R package.

For the OS, MM, and PR genomes, assemblies was performed by wtdbg2 v2.5^67^ (https://github.com/ruanjue/wtdbg2) using Oxford Nanopore long reads. The final consensus assemblies were generated after combining the polishing of the Oxford Nanopore long reads by Racon^73^ and 3 rounds polishing of the Illumina short-insert clean reads by Pilon^69^.

### Assessment of the genome assemblies

To assess the quality of genome assemblies, we used BUSCO v3^74^ to evaluated the genome completeness by estimating the percentage of expected single-copy conserved orthologues captured in our assemblies, referring to the mammalia_odb9 lineage-specific profile that contains 4,104 BUSCO gene groups.

### Genome annotation

#### Repeat annotation

Repetitive elements in the four assembled genomes were identified by a combination of homology searching and de novo prediction. RepeatMasker (version 4.0.6)^75^ and RepeatProteinMask were applied to identify repeat elements by searching against the Repbase database^76^. De novo repeat annotations were carried out by running RepeatScout v1.0.5^77^, RepeatModeler v1.0.3^78^, and LTR_FINDER v1.0.7^79^. A de novo repetitive element database was constructed and then used by RepeatMasker to annotate repeat elements. The tandem repeats were further predicted using TRF v4.07b tool^80^. The Repbase-based annotations and de novo annotations were then merged.

#### Gene annotation

Gene prediction was conducted by a combination of homology-based prediction, ab initio prediction, and RNA-seq based prediction methods. For homology-based prediction, the protein repertoires of four mammalian species downloaded from NCBI were used as queries for searches against the assembled genomes using TBLASTN^81^ (BLASTall v2.2.26) with an e-value cut-off of 1 × 10^−5^. The Basic Local Alignment Search Tool (BLAST) hits were joined together by Solar v0.9.6^82^. GeneWise v2.4.1^83^ was used to predict the exact gene structure of the corresponding genomic regions for each BLAST hit.

RNA-seq data derived from 6 tissues were assembled de novo using Trinity^84^. For OS, MM, and PR, the resulting transcripts were mapped to the genomes using the Program to Assemble Spliced Alignment (v2.0.2, PASA, https://github.com/PASApipeline/PASA-pipeline)^85^. For DS, transcripts from Trinity and transcripts from long-read RNA-seq were merged and then mapped to the DS genome using PASA. Valid transcript alignments of each species were clustered based on each genome mapping location and assembled into gene structures. Finally, RNA-seq reads were directly mapped to the genome using TopHat2^86^ to identify putative exon regions and splice junctions. Cufflinks^87^ was then used to assemble the mapped reads into gene models.

For ab initio gene prediction, Augustus v2.7^88,89^, Geneid v1.4^90^, Genescan^91^, GlimmerHMM v3.02^92^ and SNAP (28/07/2006)^93^ were used to predict coding regions in the repeat-masked genome. For Augustus, GlimmerHMM and SNAP, the high-quality gene models used for training was generated by PASA.

Gene models from three gene prediction methods were integrated by using EvidenceModeler v1.1.1 (EVM)^94^. The weights for each type of evidence were set as follows: PASA-set > Homolog-set > Cufflinks-set > Augustus > Geneid = SNAP = GlimmerHMM = GeneScan. The gene models were further updated by PASA2 to generate untranslated regions and alternative splicing variation information. Gene models supported only by homology or ab initio evidence were filtered out.

### Gene functional annotation

Functional annotation of the gene models included the annotation of functional motifs, domains, and possible related biological processes was conducted via BLASTp searches^95^ (e-value < 10^−5^) against the NR (nonredundant protein sequences in NCBI), SwissProt^96^, KEGG (Kyoto Encyclopedia of Genes and Genomes)^97^ and Gene Ontology^98^ databases and retrieved from the results of InterPro^99^.

### Phylogenetic tree construction and estimation of divergence times

In addition to the four selected species, protein sequences from 10 other species of mammals with available genomes were downloaded from the NCBI. Only the longest transcript was selected for each gene locus with alternative splicing variants. Genes containing fewer than 30 amino acids were removed. All-against-all BLASTp searches were employed to identify the similarities between the filtered protein sequences in these species, with an e-value cut-off of 1e^−7^. The OrthoMCL method^100^ was used to cluster genes from these different species into gene families with the parameter “-inflation 1.5”. The protein sequences from each family were aligned using MUSCLE^101^ with the default parameters, and gaps were trimmed using Gblocks^102^. The corresponding CDS alignments were back-translated from the corresponding protein alignments. The CDS alignments of all single-copy gene families (that is, genes with only one copy in each species in a gene family cluster) were used for further phylogenomic analyses.

For phylogenetic tree construction, CDS alignments were concatenated to generate a super alignment matrix. RAxML v8.0.19^103^ was used to reconstruct a maximum likelihood tree with 1000 rapid bootstrap iterations. Divergence times were estimated using the MCMCTREE program in the PAML 4.7^104^. These analyses involved the correlated rates (clock = 3) and JC69 models in the MCMCTREE program. Five calibration points were obtained from the TimeTree database^105^.

### Expanded and contracted gene families

Gene family expansion and contraction analyses were performed using CAFÉ^106^. First, an “expanded and contracted gene family” on each branch of the tree was detected by comparing the cluster size of each branch with the maximum likelihood cluster size of the ancestral node leading to that branch. The overall *P* value (family-wide *P*-value in CAFÉ, which is based on a Monte Carlo resampling procedure) of each branch and node was then calculated, and the exact *P* values (Viterbi method in CAFÉ) of each significant overall *P*-value (< 0.05) gene family were also calculated. Finally, for each branch and node, an “expanded and contracted gene family” with both an overall *P*-value and an exact P value < 0.05 was defined as a “significantly expanded and contracted gene family”.

### Identification of PSGs and REGs

A gene set among the 13 species on phylogenetic tree (except the outgroup *H. sapiens*) were retrieved from the OrthoMCL methodology described above. Single gene families were then extracted and the CDS from each family were aligned via MUSCLE. The resulting alignments and tree topology of phylogenetic tree were used as inputs for PSGs and REGs identification in PAML 4.7. To identify PSGs, the branch-site model was used, which allows ω to vary both among sites in the protein and across branches on the tree. The alternative model allows sites to be under positive selection on the foreground branch (ω > 1), whereas a null model limits sites to evolve neutrally or under purifying selection (ω ≤ 1). To identify REGs, the branch model was used, in which the null model (model = 0) assuming that all branches have been evolving at the same rate and the alternative model (model = 2) allowing the foreground branch to evolve under a different rate. When a focal species was performing the branch and branch-site model tests, other sequenced species were removed from background set of taxa. Likelihood ratio tests (LRTs) were used to calculate *P* values. FDR correction was used to control for multiple comparisons. To further reduce false positives in selection analysis with the branch-site model, we added a post hoc filtering step: FDR correction was less than 0.01, and at least one site identified by Bayes empirical Bayes (BEB) analysis should be with a posterior probability > 0.5.

### Convergent evolution among jerboas, gerbils, and hamsters

The same gene set from PSGs and REGs analysis was used to test for convergence among the three representative clades (jerboas, gerbils, and hamsters) in Eurasian inland. Following a recent study^28^, signals of convergent evolution were detected using two methods: JTT-Fgene model^26,107^ and the PCOC method^27^. JTT-Fgene model considered a site as convergence if amino acids of a focused node at that site are the same but different with their most recent ancestral amino acids. We reconstructed amino acid sequences of internal nodes by CODEML in PAML. Then we compared the number of observed convergent site in each gene with the neutral expectations derived from the JTT-Fgene model, followed by Poisson test to evaluate the difference. PCOC method considered shifts in amino acid preference instead of convergent substitutions^28^. PCOC model the vector of amino acid frequencies (profile) for each position and each branch. Convergent and nonconvergent models were implemented in PCOC. Under the convergent model, an evolving site on convergent branches has different profile from that of nonconvergent branches. Under the nonconvergent model, a site has a same profile for both convergent and nonconvergent branches. PCOC identifies the better fit between the two models to detect convergent sites.

### Functional enrichment analysis

To better connect the evolved genes of the four species to particular functions or pathways, as well as to test whether they have convergent evolution in some functions or pathways related to desert adaptation, we tested gene sets from expanded or contracted gene families, PSGs and REGs, and convergent genes. Specifically, we performed the functional enrichment analyses using KOBAS (KEGG Orthology Based Annotation System) 3.0^108^. GO biological processes, molecular function, and cellular component terminologies and Kyoto Encyclopedia of Genes and Genomes (KEGG) pathways (http://www.kegg.jp) were identified. We used the *Mus musculus* genome as orthology, and a Benjamini and Hochberg correction for multiple tests to correct for false discoveries rate (FDR). Only significantly (*P* < 0.05, FDR < 0.05) over-represented GO terms and KEGG pathways were biologically meaningful. Enriched biological process GO terms were summarized and visualized in REVIGO (Reduce and Visualize Gene Ontology, http://revigo.irb.hr/)^109^.

### Demographic history and climate temporal shifts

We used the pairwise sequentially Markovian coalescent (PSMC)^62^ method to estimate changes in the effective population size (Ne) of OS, MM, PR and DS based on Illumina short-insert clean reads with a 50–60 fold sequencing depth. The whole-genome diploid consensus sequences for our four individuals were generated by SAMtools and BCFtools^110^ with the parameter C50. Sites with sequencing depths < 10 and > 100 (vcfutils.pl vcf2fq -d 10 -D 100) were removed to reduce the probability of false positives. The PSMC parameters were set as follows: -N30 -t15 -r5 -p “4 + 25 * 2 + 4 + 6”. The estimated generation time and mutation rate (per base per generation) were set to 0.5 and 2.351e^−09^ for MM, 1 and 4.212e^−09^ for OS, 0.5 and 2.187e^−09^ for PR, and 1.5 and 6.319e^−09^ for DS.

To assess the climate variation during the Quaternary, we downloaded annual mean temperature and annual precipitation layers from PaleoClim database^111^ at a 2.5 min resolution, including ten periods from 787 ka to the present. We used the raster^112^ and dplyr^113^ R packages to measure the relative temporal changes of the two variables within a defined region (Lat: 33 N, 55 N; Long: 70E, 130E) that includes the distribution of the four desert species.

## Supplementary information

Supplementary Information

Description of Additional Supplementary Files

Supplementary Data 1

Supplementary Data 2

Supplementary Data 3

Supplementary Data 4

Supplementary Data 5

Supplementary Data 6

Supplementary Data 7

Supplementary Data 8

Supplementary Data 9

Supplementary Data 10

Supplementary Data 11

Supplementary Data 12

Supplementary Data 13

Supplementary Data 14

Supplementary Data 15

reporting-summary

## Data Availability

Genome assembly, raw reads data, and RNA-seq data of the four rodents have been deposited in NCBI under projection accession: PRJNA682294 (DS), PRJNA682295 (OS), PRJNA682296 (MM), and PRJNA682297 (PR). Source data of the figures are provided with this paper in Supplementary Data 15.
