# Supplementary material for: Similar adaptative mechanism but divergent demographic history of four sympatric desert rodents in Eurasian inland
Source: Commun Biol. 2023 Jan 12;6:33. doi: 10.1038/s42003-023-04415-y (PMC9837166; doi:10.1038/s42003-023-04415-y)
Supplement: Supplementary file 1 — Supplementary Information [file 42003_2023_4415_MOESM1_ESM.docx]

Supplementary Materials for

**Similar Adaptative Mechanism but Divergent Demographic History of Four Sympatric Desert Rodents in Eurasian Inland**

Jilong Cheng^1, †^, Xingwen Peng^1, 2, †^, Hong Li^3, †^, Anderson Feijó^1^, Lin Xia^1^, Georgy I. Shenbrot^4^, Deyan Ge^1^, Zhixin Wen^1^, Dehua Wang^5^, Qisen Yang^1, *^

1 Key Laboratory of Zoological Systematics and Evolution, Institute of Zoology, Chinese Academy of Sciences, Chaoyang District, Beijing, 100101, China

2 College of Life Sciences, University of Chinese Academy of Sciences, Shijingshan District, Beijing, 100049, China

3 Novogene Bioinformatics Institute, Haidian District, Beijing 100083, China

4 Mitrani Department of Desert Ecology, Jacob Blaustein Institutes for Desert Research, Ben-Gurion University of the Negev, Midreshet Ben-Gurion, Negev, 84990, Israel

5 State Key Lab of Integrated management for Pest Insects and Rodents, Institute of Zoology, Chinese Academy of Sciences, Chaoyang District, Beijing, 100101, China

^†^ These authors contributed equally.

^∗^Corresponding author. E-mail: yangqs@ioz.ac.cn, +86-10-64807225.

**Supplementary Figures**


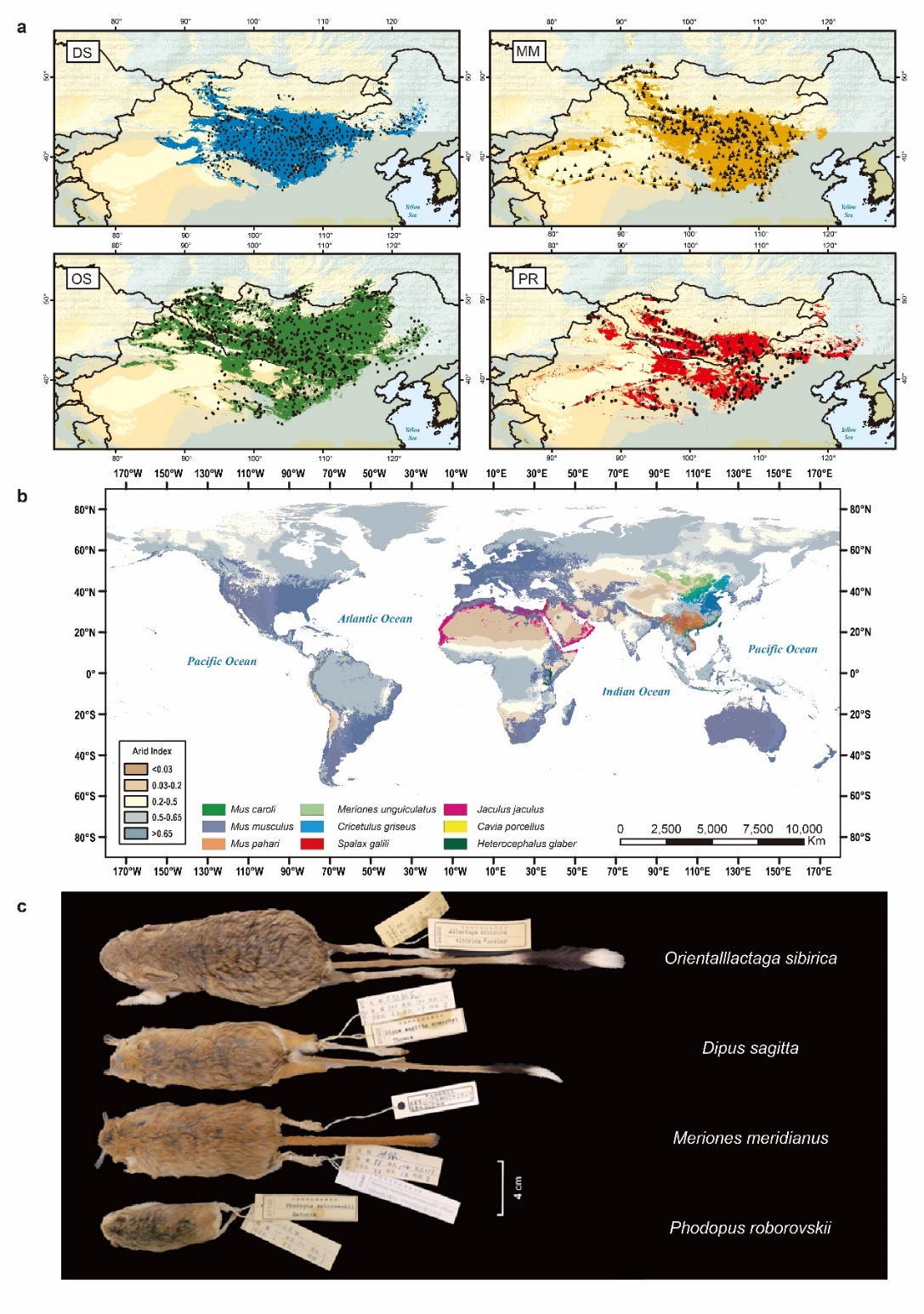


**Supplementary Figure 1. Species distribution models for species on the phylogenetic tree and specimens of the four desert rodents, *Dipus sagitta* (DS), *Orientallactaga sibirica* (OS), *Meriones meridianus* (MM), and *Phodopus roborovskii* (PR).** The distribution ranges of all species were calculated by MAXENT 3.4.0 and showed in ArcMap 10.8.1.


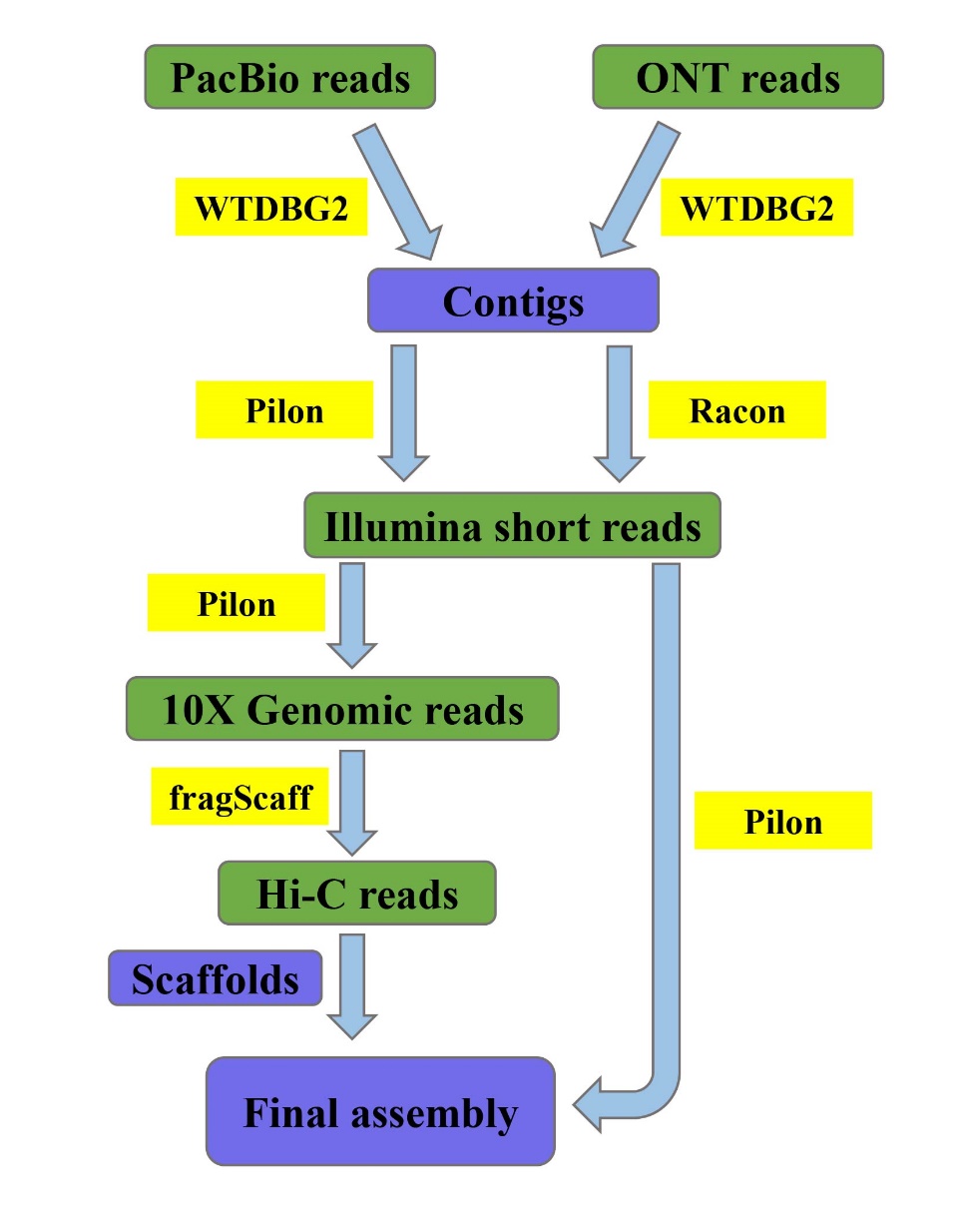


**Supplementary Figure 2. The flowchart of genome assembly in four selected desert rodents.** The *Dipus sagitta* genome was assembled and rectified with PacBio long reads, 10X genomic reads, and Hi-C reads. The genomes of *Orientallactaga sibirica,* *Meriones meridianus*, and *Phodopus roborovskii*, were assembled with Oxford Nanopore Technologies reads.


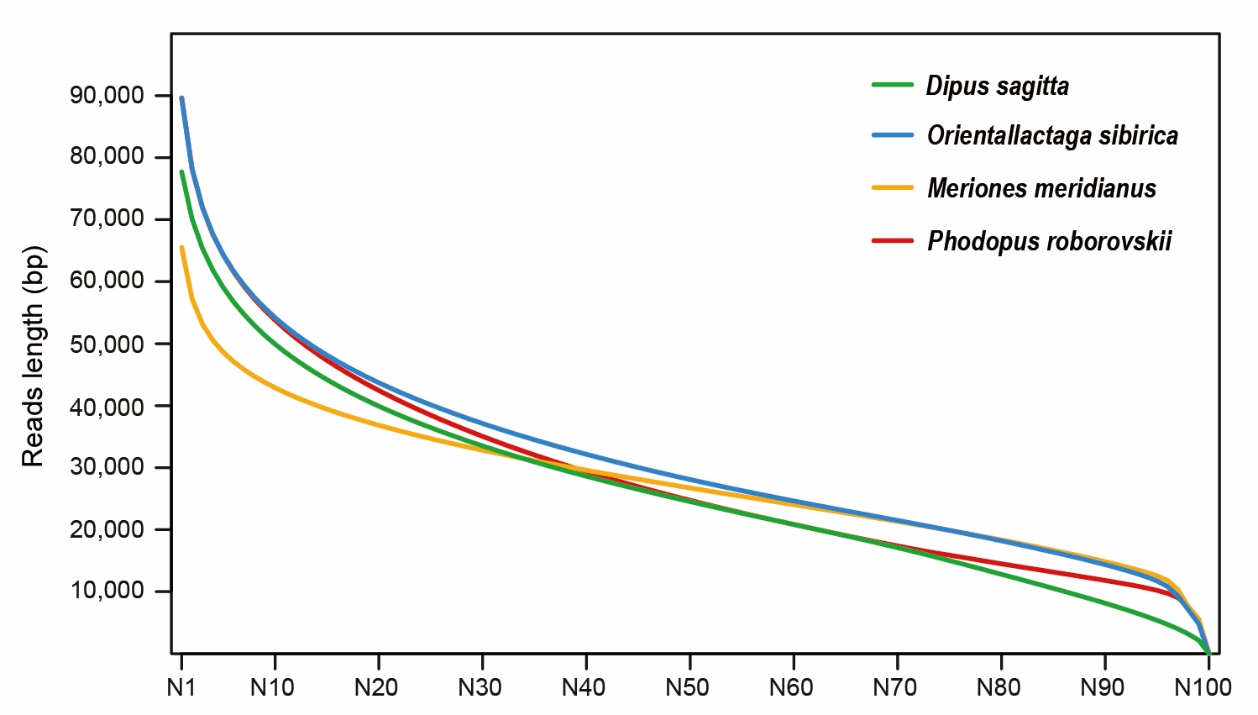


**Supplementary Figure 3.** Distribution of reads length in the selected four species.


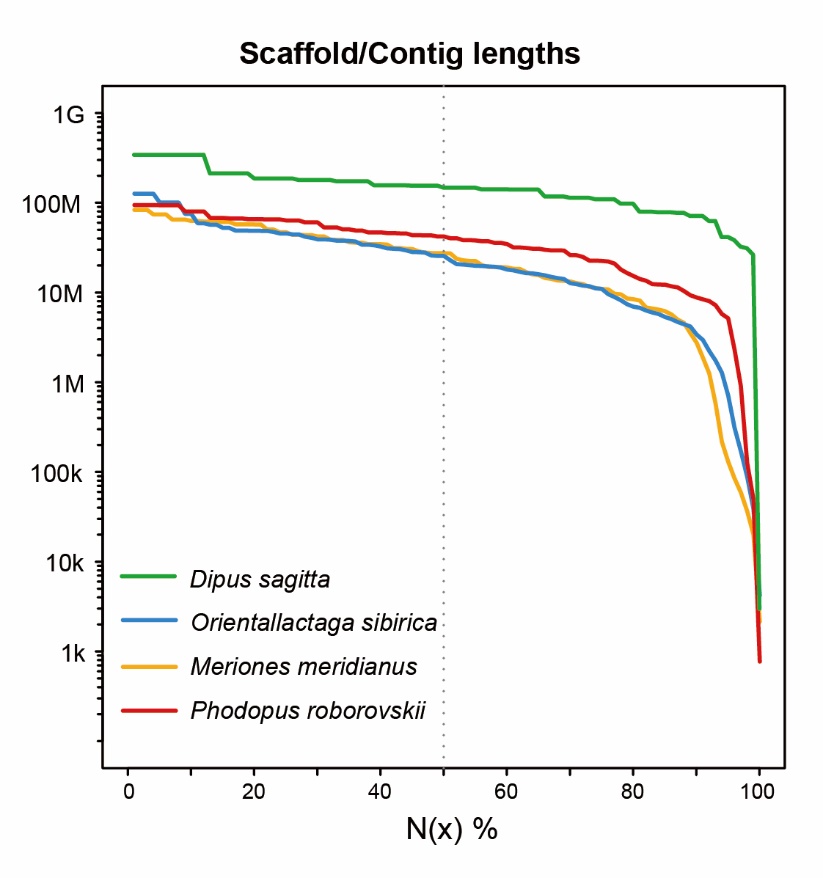


**Supplementary Figure 4.** Distribution of assembly Length in the four selected rodents.


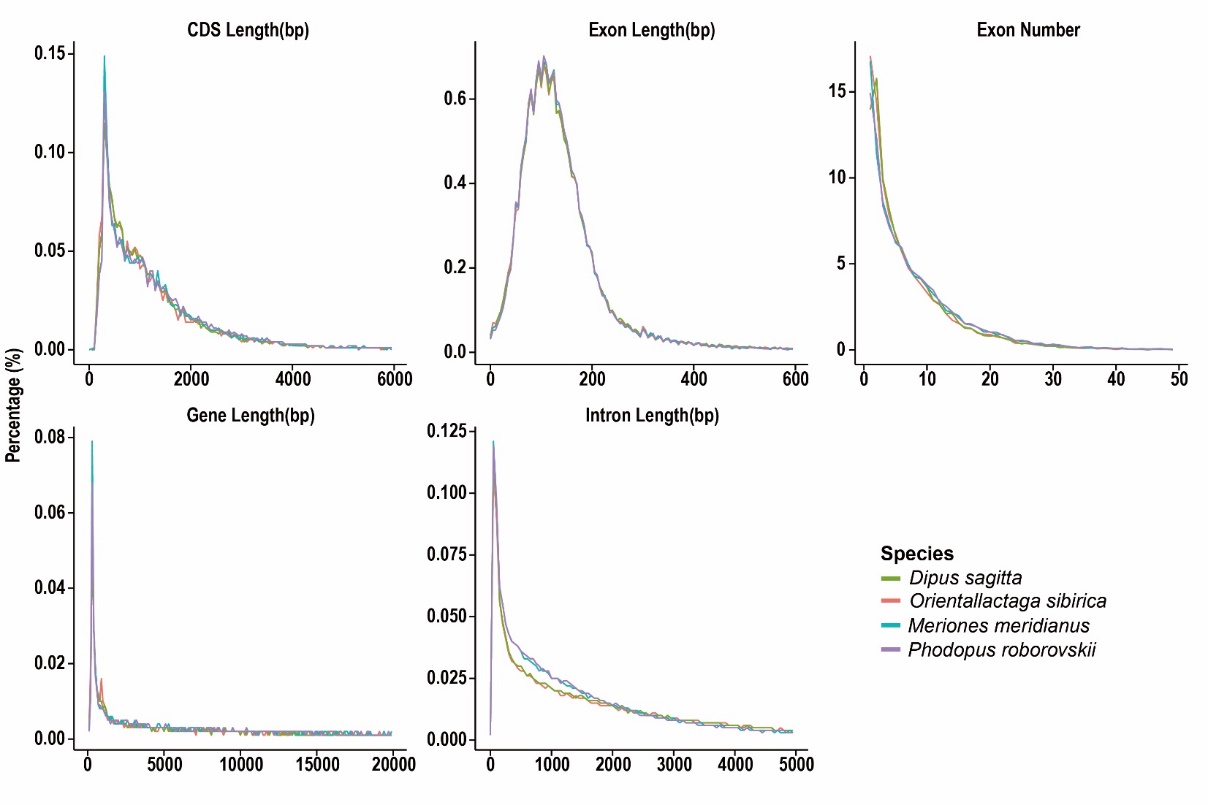


**Supplementary Figure 5. Comparison of the distribution of several features in the final gene set for the four desert rodents.** No obvious unexpected differences exist among these four organisms, indicating the high quality of gene structure annotation.


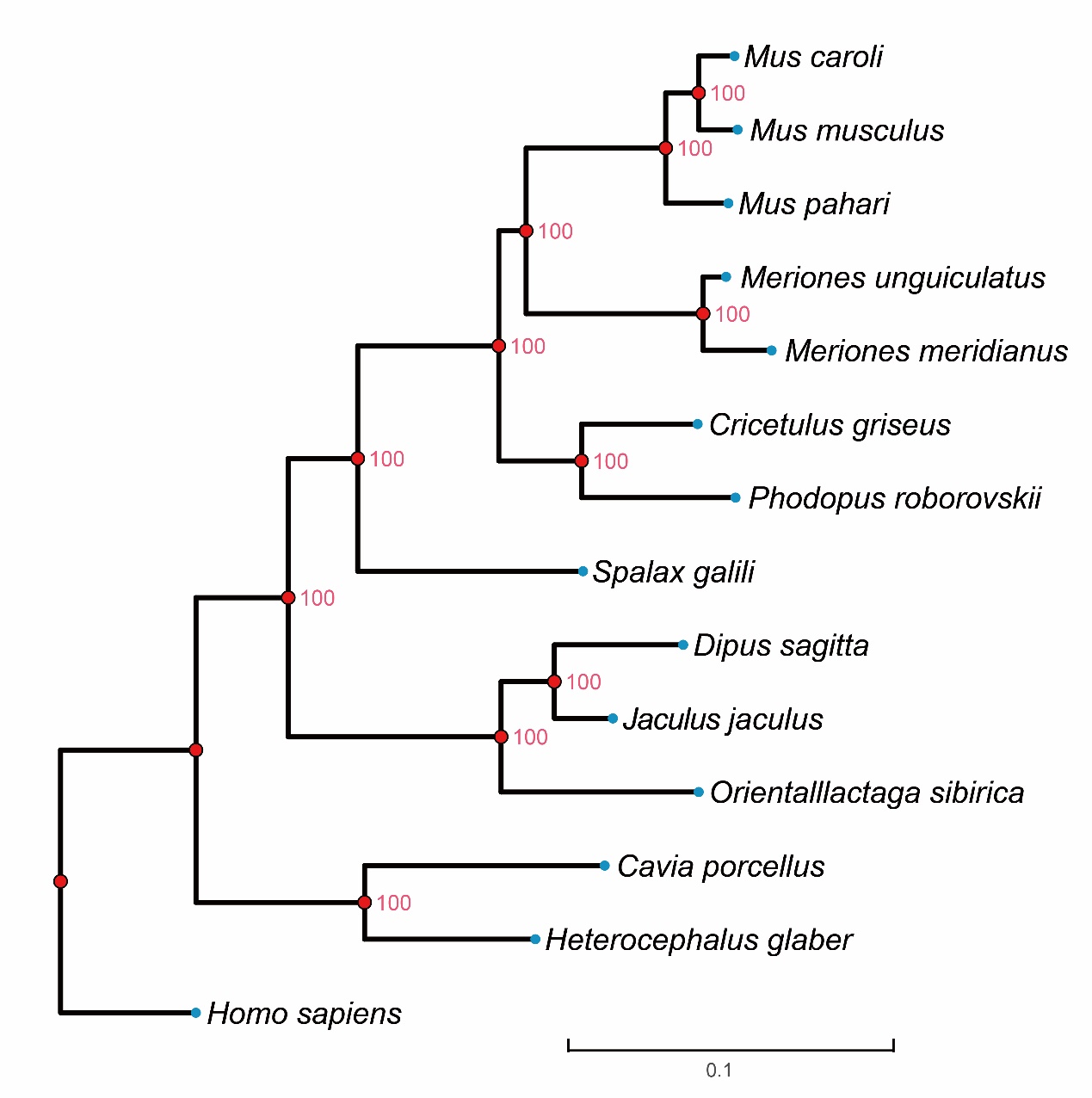


**Supplementary Figure 6. Phylogenetic tree based on 5,102 single-copy orthologous groups by using the maximum likelihood method.** Single-copy orthologous genes were from the selected genomes, including *Dipus sagitta*, *Orientallactaga sibirica*, *Meriones meridianus*, *Phodopus roborovskii*, *Cricetulus griseus* (GCF_000223135.1), *Meriones unguiculatus* (GCF_002204375.1), *Spalax galili* (GCF_000622305.1), *Mus caroli* (GCF_900094665.1), *Jaculus jaculus* (GCF_000280705.1), *Mus musculus* (GCF_000001635.26), *Mus pahari* (GCF_900095145.1), *Heterocephalus glaber* (GCF_000247695.1), *Cavia porcellus* (GCF_000151735.1), and *Homo sapiens* (GCF_000001405.39). Branch lengths represent the neutral divergence rates. Numbers on the branches represent LRT values, which illustrate the reliability of branches calculated by RAxML.


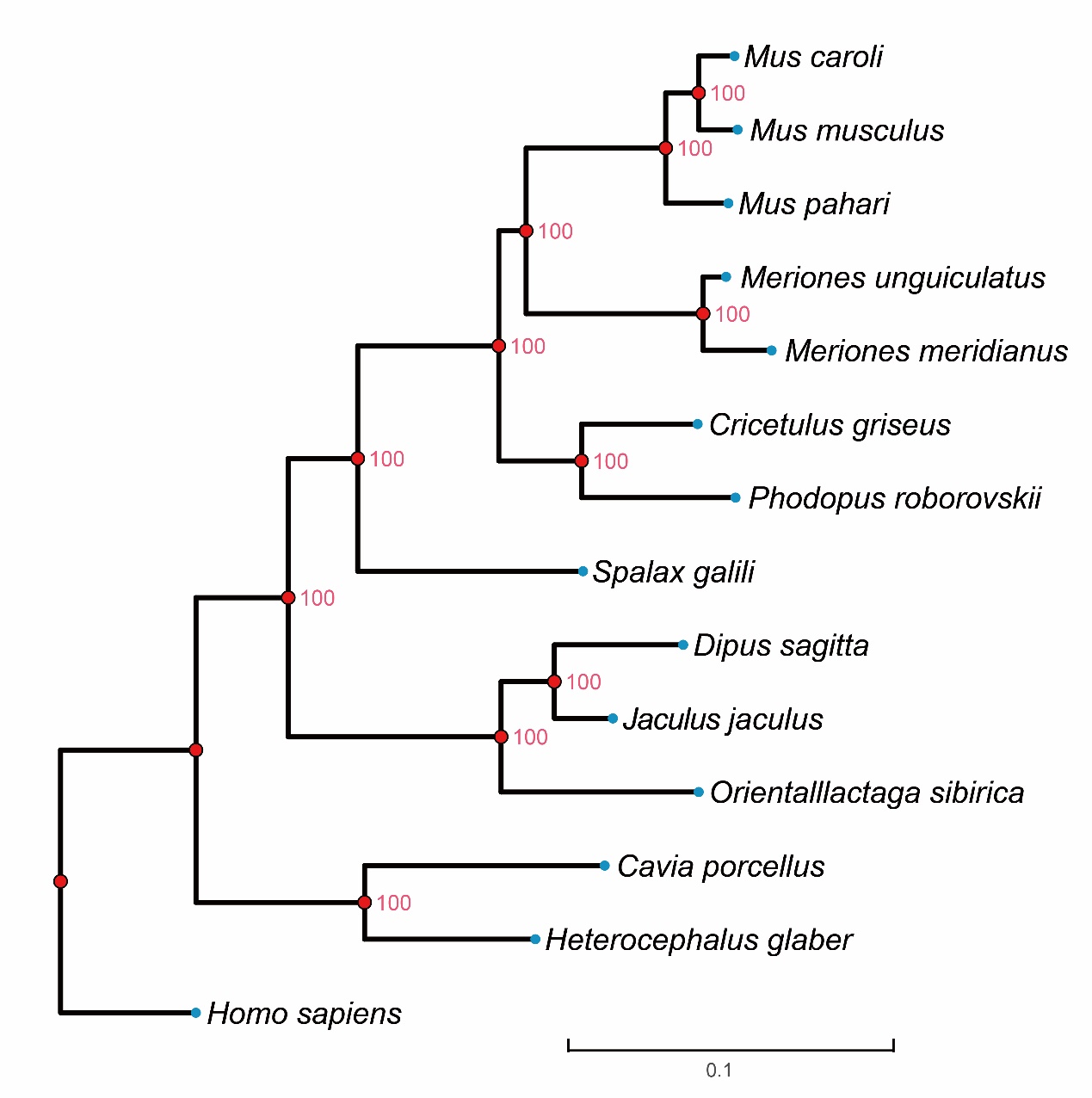


**Supplementary Figure 7. Estimation of divergence time.** The numbers on the nodes represent the divergence times from present (million years ago, Mya). The red dots in five internal nodes indicate fossil calibration times were used in the analysis. The graph showed the estimated divergence times with their 95% confidence intervals.


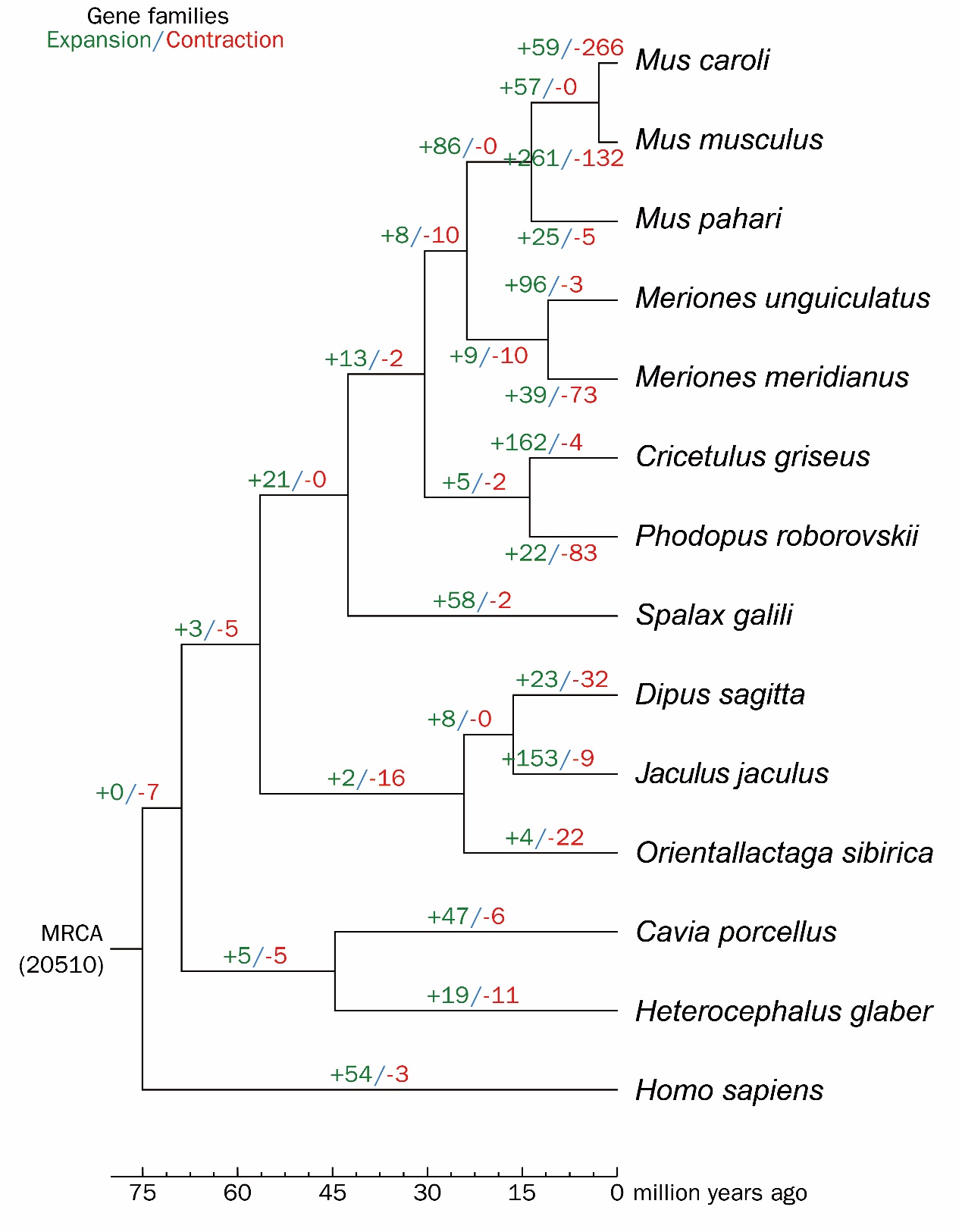


**Supplementary Figure 8.** **Expansion and contraction of gene families are shown along the phylogenetic tree.** Beside each species name are the number of gene families that underwent significant (p < 0.05) expansions (+) or contractions (-).


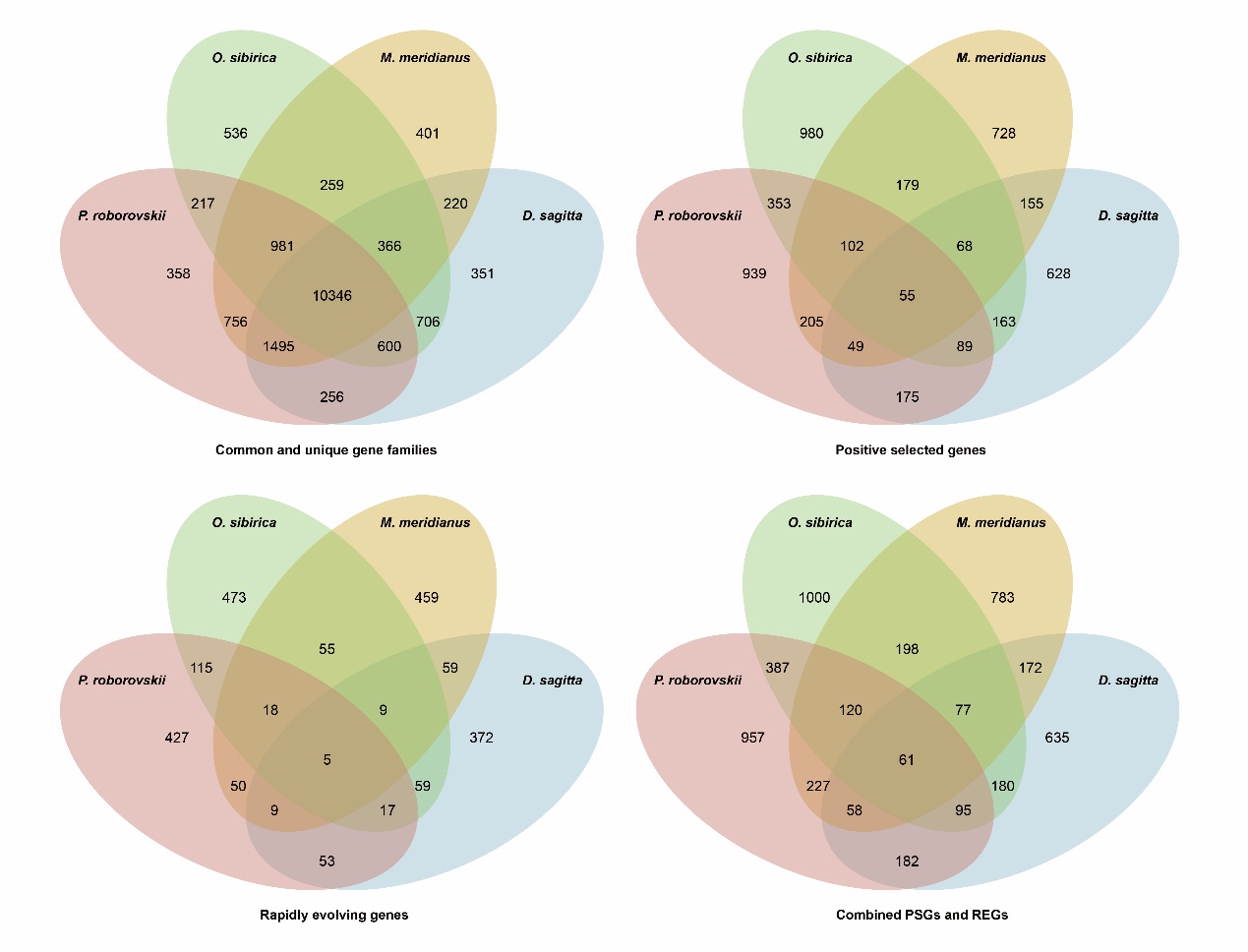
 **Supplementary Figure 9.** Comparisons of common and unique gene families, positively selected genes (PSGs), rapidly evolving genes (REGs) and combined PSGs and REGs among all four desert rodents, *Dipus sagitta*, *Orientallactaga sibirica*, *Meriones meridianus*, and *Phodopus roborovskii.*


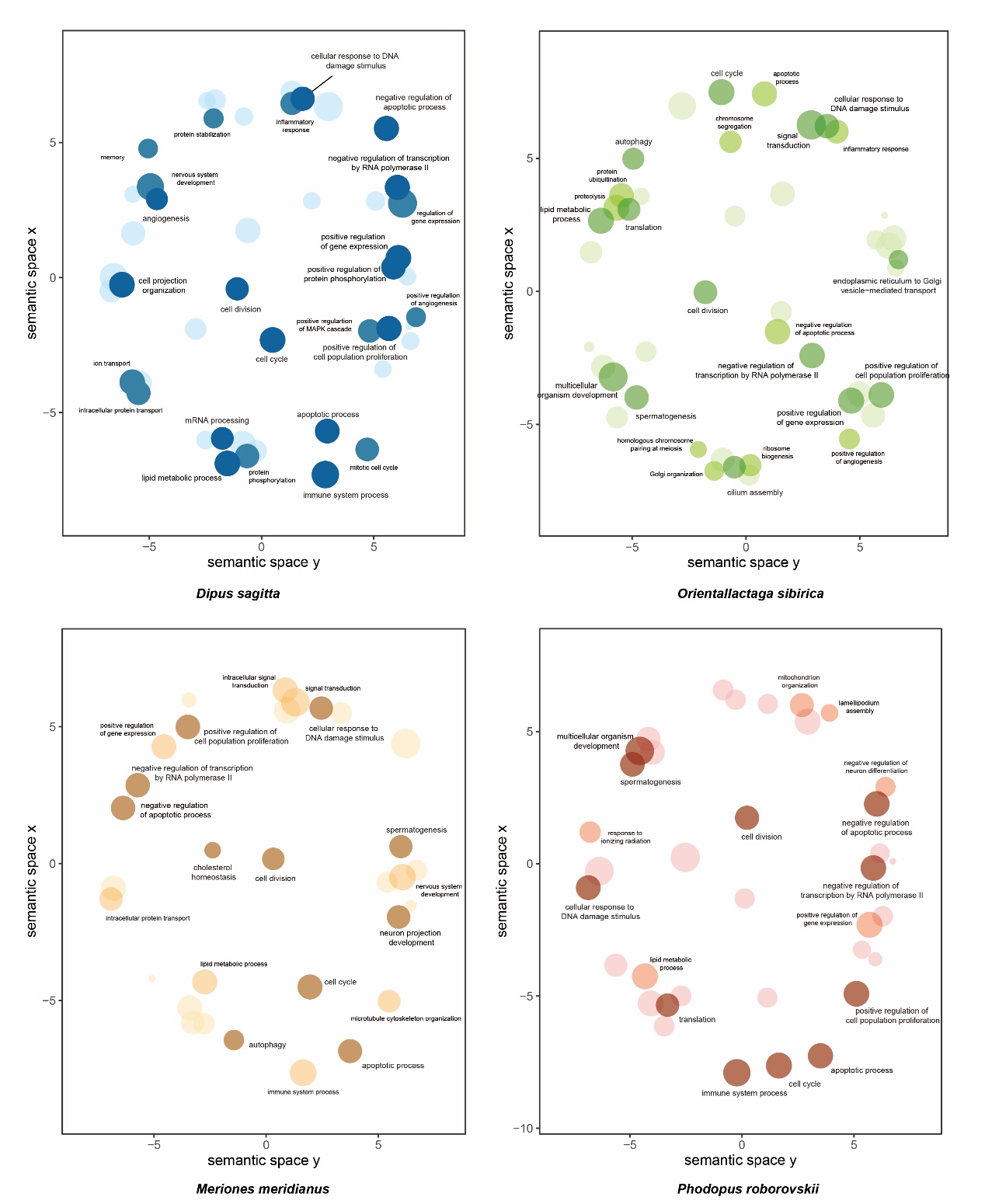


**Supplementary Figure 10. Scatterplot showing clusters representative of enriched biological process GO terms for the four desert rodents.** The color represents the size of log10 *p*-value. The darker the color, the smaller the log10 *p*-value, indicating the more significant the GO term enrichment.


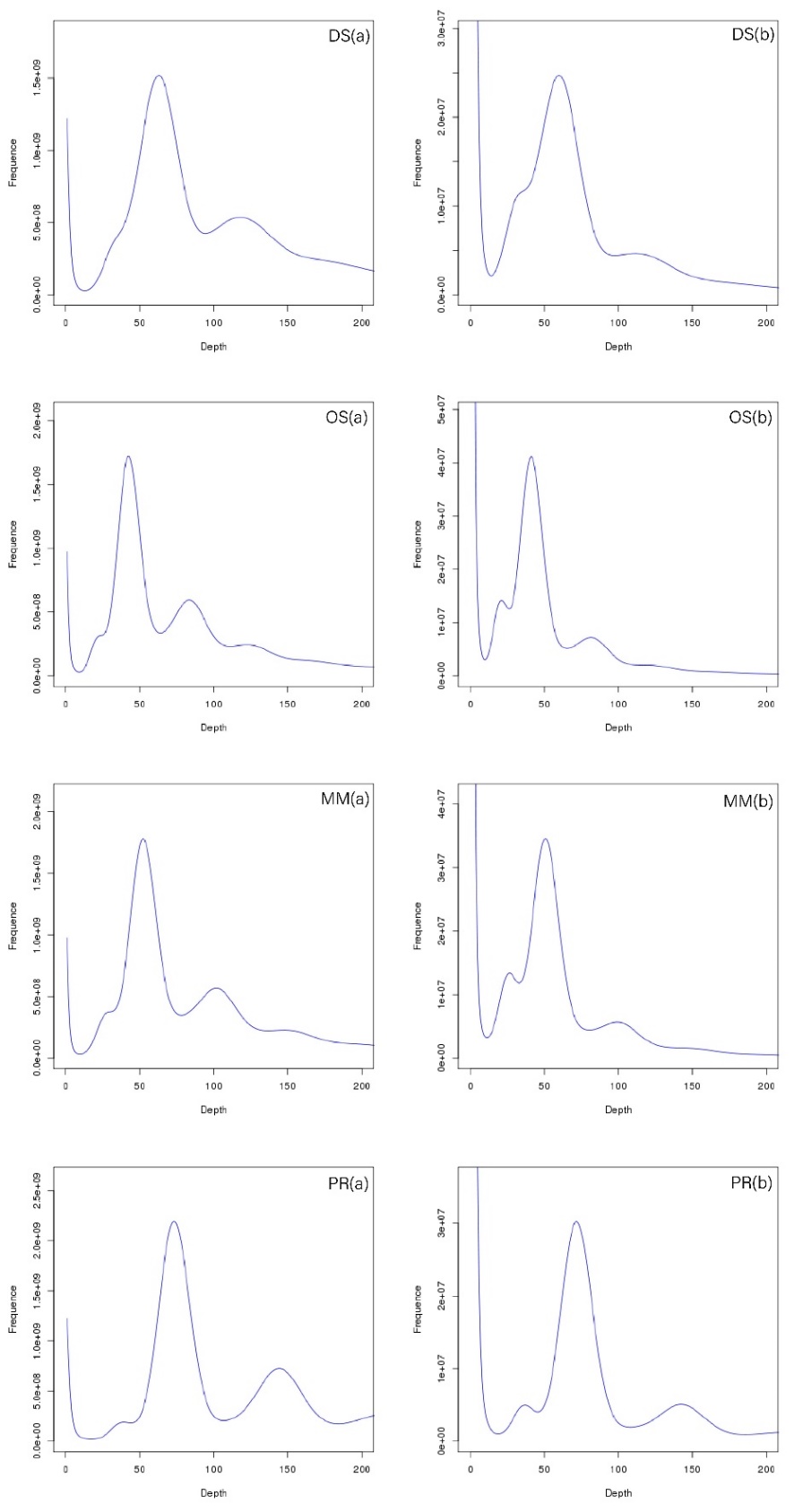


**Supplementary Figure 11.** **K-mer estimation of the genome size.** (a) Distribution of depth and K-mer number frequency, (b) Distribution of depth and K-mer category frequency. The X-axis represents the K-mer depth. The Y-axis represents the K-mer number of the corresponding depth. DS represents species *Dipus sagitta*, OS represents species *Orientallactaga sibirica*, MM represents species *Meriones meridianus*, PR represents species *Phodopus roborovskii*.

**Supplementary Tables**

**Supplementary Table 1.** Sequence production of the four desert rodents.

| **Library** | **Species** | | | |
| --- | --- | --- | --- | --- |
|  | ***Dipus sagitta*** | ***Orientallactaga sibirica*** | ***Meriones meridianus*** | ***Phodopus roborovskii*** |
| **Illumina short-insert clean reads (Gbp)** | 377.67 | 162.58 | 172.22 | 214.34 |
| **10X Genomics clean reads (Gbp)** | 304.42 | - | - | - |
| **Hi-C clean reads (Gbp)** | 389.13 | - | - | - |
| **PacBio reads (Gbp)** | 261.01 | - | - | - |
| **ONT reads (Gbp)** | - | 183.09 | 161.34 | 186.45 |
| **Sequence coverage (X)** | 474.10 | 123.45 | 137.26 | 185.55 |

**Supplementary Table 2.** Estimation of the four desert rodents' genome size using the K-mer analysis.

| **Species** | ***Dipus sagitta*** | ***Orientallactaga sibirica*** | ***Meriones meridianus*** | ***Phodopus roborovskii*** |
| --- | --- | --- | --- | --- |
| **K-mer** | 17 | 17 | 17 | 17 |
| **K-mer depth** | 62 | 41 | 51 | 72 |
| **Survey Genome size (Gbp)** | 2.91 | 3.19 | 2.61 | 2.38 |
| **Revised Genome size (Gbp)** | 2.89 | 3.17 | 2.59 | 2.36 |
| **Heterozygous rate (%)** | 0.62 | 0.46 | 0.57 | 0.28 |
| **Repeat rate (%)** | 61.13 | 65.56 | 57.18 | 55.90 |

**Supplementary Table 3.** Statistics of the genome assemblies for the four desert rodents in this study.

| **Species** | | ***Dipus sagitta*** | ***Orientallactaga sibirica*** | ***Meriones meridianus*** | ***Phodopus roborovskii*** |
| --- | --- | --- | --- | --- | --- |
| **Total** | | 2.81 Gbp | 2.83 Gbp | 2.43 Gbp | 2.16 Gbp |
| **Contig N50 (Mbp)** | | 31.41 | 25.87 | 24.08 | 42.68 |
| **Contig N90 (Mbp)** | | 5.58 | 3.44 | 2.79 | 8.91 |
| **HiC Scaffold N50 (Mbp)** | | 147.24 | - | - | - |
| **HiC Scaffold N90 (Mbp)** | | 71.23 | - | - | - |
| **BUSCO Completeness (%)** | | 93.80 | 92.90 | 95.00 | 95.90 |
| **CEGMA Completeness (%)** | **Complete** | 92.74 | 91.13 | 91.94 | 92.74 |
|  | **Complete+Partial** | 96.37 | 96.37 | 95.16 | 93.95 |
| **Sequence identity Completeness (%)** | | 99.58 | 98.27 | 97.93 | 99.46 |

**Supplementary Table 4.** Genome annotation statistics of the four desert rodents.

| **Species** | ***Dipus sagitta*** | ***Orientallactaga sibirica*** | ***Meriones meridianus*** | ***Phodopus roborovskii*** |
| --- | --- | --- | --- | --- |
| **Gene statistics** |  |  |  |  |
| Number of genes | 23,482 | 22,859 | 22,533 | 22,314 |
| CDS+intron length (bp, avg) | 33321.38 | 34235.58 | 28634.89 | 29316.51 |
| CDS length (bp, avg) | 1298.35 | 1281.37 | 1378.83 | 1435.08 |
| Exon length (bp, avg) | 176.45 | 177.32 | 170.98 | 171.42 |
| Intron length (bp, avg) | 5036.57 | 5292.63 | 3858.25 | 3782.18 |
| Exons per gene (avg) | 7.36 | 7.23 | 8.06 | 8.37 |
| **Functional annotations** |  |  |  |  |
| Genes with NR annotation [n, (%)] | 20,247 (86.2) | 19,326 (84.5) | 19,104 (84.8) | 19,178 (85.9) |
| Genes with Swissprot annotation [n, (%)] | 19,717 (84.0) | 18,841 (82.4) | 18,590 (82.5) | 18,589 (83.3) |
| Genes with KEGG annotation [n, (%)] | 17,279 (73.6) | 16,538 (72.3) | 16,418 (72.9) | 16,462 (73.8) |
| Genes with InterPro annotation [n, (%)] | 23,211 (98.8) | 22,566 (98.7) | 22,228 (98.6) | 22,014 (98.7) |
| Genes with annotated [n, (%)] | 23,267 (99.1) | 22,642 (99.1) | 22,273 (98.8) | 22,061 (98.9) |

**Supplementary Table 5.** Proportion of the genome covered by different kinds of repeat elements.

| **Species** | **Genome Size (Gb)** | **DNA** | **LINE** | | **SINE** | **LTR** | | | **Other** | **Satellite** | **Simple repeat** | **Unknown** | **Total** |
| --- | --- | --- | --- | --- | --- | --- | --- | --- | --- | --- | --- | --- | --- |
|  |  |  | **Total** | **LINE/L1** |  | **Total** | **LTR/LTR** | **LTR/ERVL** |  |  |  |  |  |
| ***Dipus sagitta*** | 2.81 | 0.02 | 7.29 | 7.26 | 0.28 | 47.39 | 43.7 | **3.43** | 0 | 0 | 0 | 0.6 | 51.17 |
| ***Orientallactaga sibirica*** | 2.83 | 0.02 | 5.14 | 5.13 | 0.24 | 50.88 | 46.9 | **3.5** | 0 | 0 | 0 | 0.15 | 53.02 |
| ***Meriones meridianus*** | 2.43 | 0.33 | 28.99 | 28.9 | 3.14 | 9.38 | 5.7 | 0.32 | 0.04 | 0.14 | 0.66 | 0.42 | 37.62 |
| ***Phodopus roborovskii*** | 2.16 | 0.25 | 7.51 | 7.49 | 1.01 | 25.14 | 21.5 | 1.04 | 0.04 | 0 | 0 | 1.14 | 31.38 |
| *Jaculus jaculus* | 2.84 | 0.03 | 6.2 | 6.19 | 0.03 | 35.46 | 32 | **3.35** | 0 | 0 | 0 | 0.55 | 39.47 |
| *Spalax galili* | 3.06 | 0.05 | 8.79 | 8.75 | 0.08 | 37.23 | 31.8 | 3.08 | 0 | 0.01 | 0.01 | 1.24 | 43.23 |
| *Meriones unguiculatus* | 2.52 | 0.18 | 10.43 | 10.4 | 0.86 | 24.76 | 22 | 0.63 | 0.09 | 0.07 | 0.01 | 2.89 | 33.16 |
| *Cricetulus griseus* | 2.4 | 0.17 | 8.51 | 8.48 | 0.45 | 28.21 | 24.6 | 1.53 | 0.05 | 0.05 | 0.05 | 1.18 | 35.38 |
| *Mus musculus* | 2.82 | 0.45 | 14.4 | 14.4 | 0.41 | 29.8 | 26.3 | 1.51 | 0.01 | 0.07 | 0.05 | 0.41 | 39.78 |
| *Mus caroli* | 2.55 | 0.04 | 8.09 | 8.07 | 0.07 | 25.41 | 22.3 | 1.7 | 0.05 | 0.02 | 0 | 0.22 | 31.09 |
| *Mus pahari* | 2.48 | 0.04 | 7.86 | 7.85 | 0.06 | 25.95 | 22.2 | 2.03 | 0.05 | 0.01 | 0.01 | 0.6 | 31.81 |

**Supplementary Table 6.** Positively selected genes (PSGs) and rapidly evolved genes (REGs) shared by all four desert rodents or any three of them.

| **Types** | **Shared species** | **Genes** |
| --- | --- | --- |
| PSG | DS/OS/MM/PR (55) | *ARHGEF3, ARMCX4, ASIC5, ATXN2, ATXN2L, CATSPER4, CCDC136, CD72, CELF5, DHX29, DLEC1, DMKN, DMTN, EIF2AK1, FCHO2, FRMD7, GPAT2, HSD17B3, KIRREL3, LRCH3, MAPKAPK3, MEI1, MGEA5, MSLNL, MUSK, NPAS4, NTN5, OGFOD3, PBRM1, PDE10A, PLCZ1, PLIN4, PRR3, PVRL3, RCAN2, RNF183, SETD1A, SGCE, SH2B1, SNAPC4, SPATA19, SPATS1, SPEF1, SPRYD3, SRCIN1, SYN3, TCTN1, TMCO3, TMPRSS7, TTLL3, UMODL1, WNT7B, ZAR1, ZNF316, ZP2* |
|  | DS/OS/MM (102) | *ACR, ANKH, ANO9, ARHGAP12, ARHGAP30, ARL14EPL, ASAH2, ATE1, ATP6V0B, BCCIP, C1QTNF4, CACNG2, CAPN7, CCAR2, CCDC37, CCDC92, CD86, CDHR2, CHKB, CPA1, DISC1, DNMT3L, DOC2G, DZIP3, EIF2AK4, EIF4E1B, EIF4G2, ELK4, ENDOU, FANK1, FOXRED1, GPM6B, GRIN1, HECA, HID1, HRG, IDH2, IL15RA, IPO11, IQSEC3, ISG20L2, IZUMO1, KCNJ10, KCNJ6, LMF1, LOXL1, LRGUK, METTL21A, MITF, MOXD1, MROH7, MTFR2, MUTYH, NCOA7, NFAM1, NLRC3, NMNAT2, NOP2, NOP58, OSGIN1, OSTF1, OTOA, OVCH2, PARVA, PDDC1, PMFBP1, POF1B, PRR29, RAD52, RALY, RBM14, RBP1, RBPJL, RD3, RNF187, RNF8, RPH3A, RPS6KL1, SCAMP5, SGSH, SLC37A3, SLC39A12, SNTN, SOBP, SORT1, SP7, SPINK5, SRRM2, SUPT20H, SWAP70, TBC1D21, TCOF1, TIMD4, TMEM136, TMEM150C, TMEM27, TMPRSS9, TXNDC2, VPRBP, ZC3H6, ZNF362, ZUFSP* |
|  | DS/OS/PR (90) | *ADAD2, ANKS6, ANP32A, ARHGAP24, ASZ1, AWAT1, CARD14, CCDC167, CCDC169, CDC14B, CHMP7, COBLL1, COL17A1, COL9A1, CSF1, CYB5R3, DFNB31, DOK2, DSPP, ELK3, EOGT, EPM2A, EVL, FAH, FAM161A, FANCI, FDFT1, FLYWCH1, FOXN4, GABRA6, GDPD4, GFRAL, GLYATL3, GRAMD1B, HTT, IFT80, IL17RD, IL1RAP, IQCE, KCNQ1, LRSAM1, MAP7, MCMBP, MED23, MPHOSPH9, MRPL4, MUT, NAA60, NFATC3, NPY5R, NR2E1, PACRG, PASK, PAX9, PIGR, PNLDC1, POM121, PYCR1, RABL3, RAD51B, RALYL, RAPGEF1, RASSF2, RBM25, RBM34, ROGDI, SDHA, SKA3, SLC8B1, SVOP, SYDE2, TELO2, TFPI, THEMIS, THOC6, TMEM179B, TOM1L2, TRIM54, VPS37A, WASF3, WFDC5, WIPI1, WNK4, XRCC4, ZBTB16, ZBTB18, ZDHHC17, ZMAT2, ZNF839* |
|  | DS/MM/PR (49) | *APP, B3GALNT2, BTG3, C8ORF88, CATSPERD, CBS, CCDC84, CCER2, CD300A, CHST11, DCX, E2F4, EEF2K, FAM64A, FBXL18, FHAD1, GPAA1, HEXDC, IL20RB, IRF2BPL, KDELC1, KIDINS220, LBP, LHX8, LRRC34, LSAMP, MIS18A, MOXD2, NDUFV3, NME8, NPAS3, ODF2L, OPCML, PAX6, PMCH, PRKAB1, RAB26, SFXN1, SLC25A26, SLK, SPACA3, STMND1, TFAP2D, TMC8, TMPRSS12, TPCN1, TPM2, TRDMT1, ZDHHC18* |
|  | OS/MM/PR (68) | *AIM1, ALG6, ARL16, BAZ1B, C1ORF127, CACNG3, CAT, CCDC162P, CDC123, CEP250, CHTF8, CIB3, CKAP2L, CMTM7, COL7A1, DHRS11, DMP1, DMTF1, ERICH6, FNDC7, FOXR1, HMGB3, HORMAD1, INVS, KLHL29, LEF1, LRRC2, MAML2, MEIKIN, MEIOB, METTL21B, MYRIP, NDUFS4, NR1D1, NUBP2, OGG1, PABPN1L, PHKB, PP2D1, PRICKLE4, PRSS22, PRSS53, RABEP2, RAI1, RFX4, RNF216, RRAS2, RS1, S100A13, SARNP, SDC2, SLMAP, SMG7, SNPH, SPERT, SYBU, TMEM19, TMEM196, TNFRSF1B, TPP2, UBXN11, UQCC2, VPS16, WDR41, WDR78, XBP1, ZFYVE19, ZNF512B* |
| REG | DS/OS/MM/PR (5) | *ARHGEF3, IL1RAP, RCAN2, SDHA, SH2B1* |
|  | DS/OS/MM (18) | *ATXN2, FBXL7, FCHO2, GPM6B, ILF2, IPO11, KCNJ6, KIRREL3, PDDC1, PDLIM3, RAB27A, RBM14, SGSH, SLC39A12, SUB1, TBC1D20, ZNF362, ZSWIM8* |
|  | DS/OS/PR (17) | *ARNTL, ELK3, EVL, L3MBTL3, MED23, MOB4, NAA60, OLA1, PACRG, PVRL3, RBM25, RBMS1, RECK, SVOP, TMCO3, WASF3, ZDHHC17* |
|  | DS/MM/PR (9) | *CELF5, DCX, GPAA1, MYLIP, NETO1, PMCH, PRKAB1, PWP2, TYW5* |
|  | OS/MM/PR (9) | *CACNG3, GABBR1, GRIN1, PXDN, RPS13, RPS2, TMEM196, TPP2, VPS16* |

**Supplementary Table 7.** Convergent genes detected by the JTT-Fgene model and PCOC method among the three representative taxa. Here we list genes that observed convergent sites in at least two pairs of comparisons in JTT-Fgene model and genes with more than 10 convergent sites detected by PCOC method.

| **Gene_name** | **hamster.vs.gerbil** | **hamster.vs.jerboa** | **gerbil.vs.jerboa** | **PCOC_0.95** | **PCOC_0.95_site** |
| --- | --- | --- | --- | --- | --- |
| *ABTB1* | + | + | + | 2 | 334, 413 |
| *SUSD4* | + | + | + | 6 | 91, 220, 268, 326, 327, 628 |
| *SYNE3* | + | + | + | 13 | 222, 330, 418, 450, 473, 512, 610, 632, 643, 879, 1014, 1019, 1051 |
| *FBLN2* | + | + | - | 15 | 101, 159, 309, 475, 482, 494, 502, 517, 519, 556, 572, 806, 929, 941, 1040 |
| *POLE* | + | + | - | 7 | 11, 785, 1203, 1776, 2044, 2197, 2303 |
| *PYROXD2* | + | + | - | 5 | 38, 63, 66, 375, 602 |
| *OLFML2B* | + | + | - | 4 | 738, 756, 777, 781 |
| *CDC42EP1* | + | + | - | 2 | 146, 176 |
| *ZNF513* | + | + | - | 1 | 568 |
| *CMIP* | + | + | - | 0 |  |
| *SESTD1* | + | + | - | 0 |  |
| *TNFRSF14* | + | - | + | 35 | 530, 532, 535, 572, 589, 629, 660, 743, 748, 756, 764, 765, 779, 808, 816, 819, 823, 845, 847, 848, 849, 876, 877, 882, 884, 885, 890, 894, 895, 896, 903, 906, 907, 909, 912 |
| *CHAT* | + | - | + | 7 | 212, 254, 606, 611, 737, 800, 830 |
| *FFAR2* | + | - | + | 7 | 158, 272, 346, 372, 398, 410, 412 |
| *MOGAT2* | + | - | + | 5 | 236, 300, 458, 460, 489 |
| *SSUH2* | + | - | + | 5 | 81, 444, 583, 612, 638 |
| *KCNK1* | + | - | + | 3 | 449, 457, 466 |
| *OTOP2* | + | - | + | 3 | 4, 232, 303 |
| *PTHLH* | + | - | + | 2 | 249, 251 |
| *WRNIP1* | + | - | + | 2 | 135, 210 |
| *SOBP* | + | - | + | 2 | 1023, 1037 |
| *RNF43* | + | - | + | 2 | 401, 696 |
| *PPM1H* | + | - | + | 1 | 306 |
| *SLC35B2* | + | - | + | 1 | 551 |
| *DLK2* | + | - | + | 0 |  |
| *ASIC4* | + | - | + | 0 |  |
| *MED27* | + | - | + | 0 |  |
| *SULT4A1* | + | - | + | 0 |  |
| *TMEM203* | + | - | + | 0 |  |
| *SLC19A1* | - | + | + | 14 | 152, 399, 410, 418, 448, 450, 474, 511, 571, 607, 614, 638, 658, 671 |
| *SLC34A3* | - | + | + | 9 | 109, 235, 270, 304, 328, 335, 359, 664, 674 |
| *KISS1R* | - | + | + | 7 | 129, 130, 134, 161, 552, 576, 582 |
| *ADAT1* | - | + | + | 6 | 102, 193, 196, 332, 497, 536 |
| *PAXX* | - | + | + | 6 | 47, 85, 91, 99, 177, 210 |
| *ZDHHC24* | - | + | + | 5 | 112, 117, 118, 126, 347 |
| *SHISA8* | - | + | + | 5 | 24, 27, 109, 564, 573 |
| *PKMYT1* | - | + | + | 3 | 141, 355, 615 |
| *TM4SF4* | - | + | + | 2 | 5, 125 |
| *CD68* | - | + | + | 2 | 45, 376 |
| *RHBDL3* | - | + | + | 2 | 31, 86 |
| *TMEM129* | - | + | + | 2 | 217, 241 |
| *PBRM1* | - | + | + | 1 | 1524 |
| *TMEM98* | - | + | + | 1 | 40 |
| *CYGB* | - | + | + | 0 |  |
| *HNRNPM* | - | + | + | 0 |  |
| *PIGS* | - | + | + | 0 |  |
| *SOX13* | - | + | + | 0 |  |
| *MAP1S* | - | + | - | 43 | 11, 200, 209, 225, 243, 366, 400, 408, 428, 473, 478, 500, 519, 522, 542, 544, 545, 556, 564, 580, 601, 602, 603, 622, 623, 634, 636, 643, 648, 650, 655, 664, 671, 677, 679, 705, 710, 712, 716, 869, 930, 1025, 1047 |
| *TUBGCP6* | + | - | - | 39 | 69, 434, 498, 627, 915, 922, 923, 927, 938, 941, 949, 955, 960, 962, 965, 966, 1025, 1032, 1053, 1080, 1081, 1093, 1094, 1116, 1119, 1122, 1128, 1191, 1362, 1427, 1429, 1433, 1447, 1453, 1462, 1490, 1506, 1507, 1536 |
| *SOHLH1* | - | + | - | 33 | 29, 34, 37, 44, 45, 46, 66, 74, 96, 98, 114, 125, 140, 171, 173, 191, 239, 257, 258, 272, 282, 283, 285, 312, 320, 338, 344, 360, 372, 378, 420, 442, 470 |
| *MMRN2* | - | + | - | 32 | 145, 198, 212, 235, 287, 311, 327, 329, 350, 353, 373, 377, 390, 457, 478, 500, 583, 702, 723, 725, 747, 760, 775, 796, 800, 813, 816, 892, 925, 983, 1004, 1092 |
| *KIAA1755* | - | + | - | 30 | 136, 212, 252, 309, 367, 397, 406, 466, 477, 503, 508, 515, 566, 680, 701, 753, 823, 902, 1010, 1059, 1063, 1083, 1085, 1092, 1103, 1106, 1117, 1141, 1155, 1169 |
| *VWCE* | - | + | - | 29 | 66, 72, 94, 400, 448, 564, 578, 585, 593, 641, 829, 856, 876, 907, 922, 928, 932, 940, 942, 955, 968, 984, 994, 996, 1002, 1018, 1045, 1051, 1066 |
| *CTC1* | + | - | - | 24 | 48, 82, 123, 241, 260, 262, 288, 329, 383, 402, 506, 514, 650, 734, 782, 796, 818, 819, 820, 858, 987, 1212, 1248, 1306 |
| *CSF3R* | - | + | - | 22 | 372, 400, 404, 410, 429, 479, 517, 593, 595, 608, 635, 656, 684, 686, 690, 823, 919, 989, 1050, 1111, 1142, 1174 |
| *TAF1C* | + | - | - | 21 | 131, 145, 324, 430, 555, 624, 643, 657, 686, 698, 725, 734, 735, 738, 792, 837, 857, 886, 898, 951, 984 |
| *PRRT3* | - | - | + | 20 | 32, 101, 118, 121, 151, 152, 219, 231, 260, 293, 320, 352, 378, 440, 630, 636, 818, 843, 990, 1021 |
| *LPAR5* | - | - | + | 19 | 62, 67, 71, 161, 217, 231, 232, 242, 243, 245, 281, 289, 300, 403, 406, 420, 421, 423, 446 |
| *SELP* | - | + | - | 19 | 42, 124, 141, 166, 185, 252, 346, 358, 384, 516, 583, 604, 608, 648, 650, 652, 790, 840, 874 |
| *MTBP* | + | - | - | 19 | 32, 338, 410, 412, 444, 487, 508, 522, 530, 542, 557, 801, 806, 850, 859, 864, 869, 880, 891 |
| *CGN* | - | - | + | 19 | 249, 250, 285, 343, 345, 383, 422, 430, 570, 634, 722, 790, 806, 846, 847, 865, 882, 885, 907 |
| *BRAT1* | - | - | + | 18 | 133, 231, 262, 272, 294, 410, 493, 516, 614, 662, 684, 788, 817, 864, 885, 938, 960, 969 |
| *BTNL9* | - | - | + | 17 | 34, 43, 60, 95, 100, 110, 112, 222, 282, 328, 370, 402, 491, 550, 554, 570, 574 |
| *MST1* | - | + | - | 16 | 27, 65, 110, 111, 216, 354, 360, 373, 448, 521, 523, 563, 568, 690, 705, 754 |
| *PRLR* | + | - | - | 16 | 40, 77, 235, 271, 285, 315, 333, 394, 414, 620, 639, 642, 689, 707, 757, 781 |
| *LAG3* | + | - | - | 15 | 12, 86, 102, 226, 256, 311, 325, 326, 406, 408, 446, 447, 468, 534, 553 |
| *CATSPER3* | - | - | + | 15 | 69, 73, 77, 97, 104, 131, 141, 142, 211, 388, 428, 475, 496, 503, 584 |
| *FAM83C* | - | + | - | 15 | 394, 486, 498, 574, 580, 596, 609, 612, 619, 626, 642, 649, 757, 758, 783 |
| *SLC8B1* | - | - | + | 14 | 118, 171, 224, 387, 431, 512, 525, 536, 584, 747, 796, 827, 831, 844 |
| *KANK2* | - | + | - | 14 | 355, 389, 415, 420, 438, 475, 476, 485, 611, 614, 619, 633, 634, 812 |
| *MYLK* | + | - | - | 14 | 237, 274, 437, 461, 464, 466, 524, 562, 633, 679, 733, 792, 1165, 1723 |
| *TTC22* | + | - | - | 14 | 137, 208, 244, 252, 322, 371, 404, 433, 551, 595, 666, 668, 672, 675 |
| *EPHX3* | - | - | + | 14 | 8, 11, 69, 79, 87, 89, 104, 115, 253, 341, 345, 409, 413, 416 |
| *FANCF* | - | + | - | 14 | 48, 72, 93, 94, 141, 152, 179, 211, 221, 227, 240, 279, 360, 398 |
| *GALC* | - | + | - | 14 | 278, 290, 322, 528, 564, 593, 595, 667, 669, 697, 715, 716, 720, 731 |
| *TOR4A* | + | - | - | 13 | 6, 51, 70, 87, 114, 228, 321, 327, 331, 332, 333, 344, 350 |
| *ENTHD2* | - | + | - | 13 | 518, 616, 705, 753, 829, 847, 852, 854, 865, 866, 915, 920, 923 |
| *PPP1R12C* | - | - | + | 13 | 9, 101, 102, 457, 490, 526, 544, 549, 605, 673, 675, 684, 721 |
| *FAAP100* | + | - | - | 13 | 4, 106, 117, 170, 302, 309, 374, 404, 565, 571, 665, 784, 951 |
| *GJD4* | - | - | + | 13 | 40, 110, 126, 157, 252, 270, 289, 290, 296, 322, 358, 363, 392 |
| *TCTE1* | - | - | + | 13 | 9, 35, 42, 50, 51, 56, 134, 231, 359, 448, 494, 503, 507 |
| *IL17RB* | - | + | - | 12 | 233, 273, 410, 438, 455, 519, 538, 671, 679, 709, 711, 720 |
| *R3HDML* | - | + | - | 12 | 62, 108, 117, 118, 138, 150, 152, 189, 210, 224, 238, 266 |
| *LMF2* | + | - | - | 12 | 192, 268, 272, 405, 505, 647, 650, 653, 704, 744, 784, 794 |
| *CDH16* | - | + | - | 12 | 156, 186, 313, 386, 476, 478, 569, 611, 791, 818, 924, 986 |
| *TMC8* | - | + | - | 12 | 79, 115, 135, 378, 407, 410, 453, 485, 897, 950, 998, 999 |
| *MED26* | - | - | + | 12 | 144, 148, 154, 164, 199, 246, 262, 272, 331, 564, 570, 584 |
| *PIGZ* | - | + | - | 12 | 233, 314, 357, 365, 375, 385, 469, 484, 521, 556, 645, 659 |
| *C19ORF68* | - | + | - | 12 | 41, 127, 128, 486, 505, 513, 562, 563, 587, 675, 695, 838 |
| *ZBTB40* | - | + | - | 12 | 256, 276, 351, 389, 390, 465, 485, 500, 642, 674, 737, 785 |
| *TET3* | - | + | - | 11 | 64, 449, 589, 688, 699, 1272, 1479, 1618, 1692, 1706, 1829 |
| *BATF2* | - | - | + | 11 | 189, 201, 212, 219, 225, 237, 247, 253, 271, 332, 351 |
| *WDR6* | + | - | - | 11 | 306, 640, 656, 671, 804, 906, 913, 948, 997, 1078, 1165 |
| *ZNF185* | - | - | + | 11 | 362, 483, 591, 833, 932, 952, 991, 1023, 1054, 1071, 1081 |
| *ARID5A* | + | - | - | 11 | 424, 433, 449, 470, 508, 534, 571, 572, 575, 583, 616 |
| *CCDC151* | + | - | - | 11 | 63, 81, 82, 201, 272, 285, 413, 470, 509, 558, 572 |
| *PALD1* | + | - | - | 11 | 24, 118, 129, 309, 311, 426, 444, 618, 629, 792, 877 |
| *PODXL2* | - | + | - | 11 | 113, 235, 319, 342, 373, 397, 404, 413, 423, 442, 446 |
| *COASY* | + | - | - | 11 | 83, 87, 115, 169, 171, 316, 351, 397, 416, 512, 574 |
| *SETBP1* | - | - | + | 11 | 344, 421, 488, 505, 512, 515, 771, 1234, 1581, 1591, 1593 |
| *PLA2G2F* | - | + | - | 8 | 122, 218, 259, 265, 299, 300, 307, 308 |
| *PTGDS* | - | - | + | 8 | 161, 162, 227, 234, 262, 272, 278, 325 |

**Supplementary Note 1**

**Phylogeny and Historical Biogeography of Jerboas, Gerbils and Hamsters**

Jerboas belong to Dipodidae, Dipodoidea ^1^. Dipodoidea shows three ecomorphotypes: Birch mice (Sminthidae), Jumping mice (Zapodidae), and Jerboas (Dipodidae) ^2-4^. Birch mice mainly found in the subalpine meadows and the boreal and alpine forests of Europe, Russia and Central and Eastern Asia. Jumping mice typically inhabit riparian or wooded areas and marshlands within coniferous forests in North America and China. Jerboas are distributed in the deserts, semi-deserts and steppes of North Africa and Eurasia. The divergence between Dipodoidea (including Jerboas) and Muroidea (including Gerbils and Hamsters) occurred in the late Palaeocene and modern Dipodoidea diversified during the middle Eocene ^3,4^. The reconstruction of ancestral areas and biogeographical events indicated that modern Dipodoidea originated in the Himalaya-Tibetan and Central Asian region ^4^. Zhang et al. (2013) indicated that zapodines and jerboas were derived from ancestral sicistines after the period of significant global cooling (since Late Miocene) ^3^. Although no ancestral state reconstruction analysis of Jerboas was performed, the fossil record, phylogenetic relationships, and evolutionary history of Dipodoidea could infer that jerboas have colonized the desert niches from their early ancestors which adapted to relatively humid and forested environment ^5^.

Gerbils belong to Gerbillinae, Muridae, Muroidea; and Hamsters belong to Cricetinae, Cricetidae, Muroidea ^1^. The divergence between Muridae and Cricetidae occurred at the Oligocene/Miocene boundary (~23Ma) ^6^. The basal divergence of Cricetidae lineages into five subfamilies occurred rapidly: hamsters (Cricetinae), voles and lemmings (Arvicolinae), Tylomyinae, Neotominae, and Sigmodontinae. Support was moderate for the basal split separating the ancestrally Old World Cricetinae+Arvicolinae clade from the endemic New World subfamilies. Muridae consisted of a basal split between the highly diverse subfamily of Old-World mice and rats, Murinae, and the remaining three subfamilies. These included the monotypic giant maned rats (Lophiomyinae), the gerbils (Gerbillinae), and the spiny mice and relatives (Deomyinae).

Ding (2020) reconstructed phylogenetic relationships of Cricetinae using mitochondrial and nuclear genes and estimated the divergence time within Cricetinae ^7^. The results indicated that the divergence time of the most recent common ancestor can be dated back to the Late Miocene for Cricetinae ^7,8^. Biogeographical analysis indicated that the extant hamsters originated in the Qinghai-Tibetan Plateau ^8^. Chevret and Dobigny (2005) estimated the divergence time between Gerbillinae and their closest relatives Acomyinae at around 17 Ma ^9^, which is in good agreement with the known gerbilline fossil record. They also supported that Gerbillinae originated in African and subsequent migrated to Asia.

Although all the three taxa (Jerboas, Gerbils and Hamsters) belong to suborder MYOMORPHA, these three groups have sister groups that are not adapted to desert habitats and diverged at least before the Oligocene/Miocene boundary (~23Ma). Moreover, derived desert specialization is considered as an “evolutionary dead end” that limits further evolution even if their ancestral state reconstructions showed marginal support for the ancestral habitat of rodents being desert ^10^. That is, we suppose if the common ancestor of all three groups (Jerboas, Gerbils, and Hamsters) had been species adapted to desert habitats, it would have been difficult for that common ancestor to have evolved more than a quarter of all extant mammals. Therefore, we consider that Jerboas, Gerbils and Hamsters have been independently colonized the desert niches since the Late Miocene.

**Supplementary Note 2**

**Species Distribution Modeling, Spatial Climate Segregation and Niche Width**

We first built occurrence points data sets for the four species used in the following analysis. The occurrence records were obtained from our field observations, the Zoological Museum of Moscow State University collections, the National Zoological Museum (Institute of Zoology, Chinese Academy of Sciences), other collections obtained using the Global Biodiversity Information Facility (GBIF, https://www.gbif.org) and publications. In total, 620 points were used for the Sowerbyi group in *Dipus sagitta* complex (DS), 1028 points for *Orientallctaga sibirica* (OS), 581 points for *Meriones psammophilus* in *Meriones meridianus* complex (MM) and 332 points for *Phodopus roborovskii* (PR).

Environmental data for species distribution modelling (SDM) were used in the form of 30 arc-second grids (approximately 1 km resolution) and were represented by climate, relief and vegetation variables. The climate variables (BioClim 1–19) were obtained from WORLDCLIM Version 2.0 ^11^, including AMT (annual mean temperature), DTR (mean diurnal range), ISO (isothermality), TS (temperature seasonality), MTWM (max temperature of warmest month), MTCM (min temperature of coldest month), TAR (temperature annual range), TEQ (mean temperature of wettest quarter), TDQ (mean temperature of driest quarter), TAQ (mean temperature of warmest quarter), TCQ (mean temperature of coldest quarter), AP (annual precipitation), PWM (precipitation of wettest month), PDM (precipitation of driest month), PS (precipitation seasonality), PEQ (precipitation of wettest quarter), PDQ (precipitation of driest quarter), PAQ (precipitation of warmest quarter), and PCQ (precipitation of coldest quarter). Altitude (ALT) data were extracted from the GOTOPO30 dataset distributed by ArcGIS. Slope data (SLOPE) were derived from the altitude layer using the Spatial Analyst module of ArcMap. Data on the Normalized Difference Vegetation Index (NDVI) were obtained from the VEGETATION Programme (<http://www.vito-eodata.be>), including data from 1998–2007 in ten-day estimates and averaged by season (winter, spring, summer, and autumn) across all available years. The NDVI is an index of greenness that is directly correlated with productivity and green vegetation biomass and is widely used in ecological studies ^12^.

The SDMs were built using MAXENT 3.4.0 software ^13^. The extent of the study area or "landscape of interest" significantly affects SDM results ^14,15^. To define the study area of a species, the kernel density of occurrence points of the species was calculated with a search radius of 4°. Then, the obtained raster was reclassified such that the original kernel density values equal to or greater than 0.001 were converted to 1, and values under 0.001 were converted to "NoData". This reclassified raster was used as the mask for clipping environmental variables to the study area. The model was constructed with the default MAXENT settings, as these settings were demonstrated to be the most appropriate for wide-ranging data ^16^. The MAXENT logistic output, which provides estimates of relative habitat suitability ^15^, was used. Most environmental variables are intercorrelated, and using all variables in an analysis can lead to an overpredicted model. To avoid overprediction, we calculated the matrix of pairwise correlations of 2 relief (altitude and slope), 19 bioclimatic and 4 NDVI variables for the study area using the “Band Collection Statistics” function in the Spatial Analyst Tools in the standard Arc Toolbox (ArcGIS Desktop 10.8.1). From the obtained correlation matrix, we chose the highest value among the correlation coefficients, built two models with one of the two mostly highly correlated variables alternately removed, estimated the model performance using the area under the receiver operating characteristic curve (AUC) test, and removed the variable whose presence in the model provided a lower AUC value from the dataset. This operation was repeated with the subsequent pairs of highly correlated variables and was stopped when the removal of a variable from the model resulted in an abrupt decline in the AUC value. The resulting model with a reduced set of environmental variables was accepted as the final model.

To delineate areas of real species occurrence, each of the original model values ranging continuously from 0 to 1 were transformed to a binary 0 or 1 using a threshold value. The threshold value was chosen as the “maximum training sensitivity plus specificity”, as it was demonstrated experimentally ^17^ that this threshold provides optimal results. All map operations were performed using ArcMap 10.8.1 software.

We compared the contributions of environmental factors to SDMs among the four species. We also evaluated which set of environmental variables were most closely associated with species distribution via principal component analysis.

The habitat niche width refers to the diversity of habitats used by a species population ^18^. The measurement of the niche width can be based on estimations of the diversity of quantitative environmental variables using diversity indices. However, this measure is appropriate only if resources are distributed evenly; otherwise, it will produce biased estimates ^19,20^. To consider uneven resource distributions, Feinsinger, et al. ^21^ proposed quantifying niche widths using the proportional similarity index (PSI), which measures the similarity between the frequency distribution of resources used by individuals of a population and the frequency distribution of resources available to them. However, the PSI cannot be used directly to compare niche widths within and among species in areas differing in the frequency distribution of resources. To estimate the unbiased niche width, we compared the similarity between the frequency distribution of resources used by individuals in a sample and the frequency distribution of resources available across the distribution range of all four species.

To eliminate the influence of factor intercorrelation, original environmental variables were normalized and then ordinated by the principal components analysis (PCA) using the Spatial Analyst module of ArcGIS. The first two principal components had eigenvalues greater than 1 and cumulatively explained 55.6% of the observed variation. The first principal component was correlated mainly with sand cover and temperature, whereas the second principal component was correlated with the NDVI and precipitation variables. These principal components were used as environmental variables. The niche breadth was estimated in the space of the first two principal components of environmental variables using kernel smoothing of the densities of species occurrence points ^23,24^. We calculated the niche breadth as the overlap between the distribution of environmental values for smoothed kernel smoothed densities of species occurrence points and the distribution of environmental values in the background environment using the *D* metric ^24^ as $D=1-\frac{1}{2}\left( \sum_{xy} \left| p_{sxy}-p_{exy} \right| \right)$, where *p_sxy_* is the proportion of the species record density at the point with coordinates *x, y* in a two-dimensional space constructed by the first two principal environmental components relative to the sum of densities at all points in this space and *p_exy_* is the frequency of environmental conditions at the point with coordinates *x, y* in a two-dimensional space constructed by the first two principal environmental components.

**Supplementary References**

1 Michaux, J. & Shenbrot, G. Dipodoidea. *Handbook of the mammals of the world, volume 7: Rodent II* (Barcelona: Lynx Ediciones, 2017).

2 Lebedev, V. S. et al. Molecular phylogeny and systematics of Dipodoidea: a test of morphology-based hypotheses. *Zool. Scr.* **42**, 231-249 (2013).

3 Zhang, Q. et al. Tracing the Origin and Diversification of Dipodoidea (Order: Rodentia): Evidence from Fossil Record and Molecular Phylogeny. *Evol. Biol.* **40**, 32-44 (2013).

4 Pisano, J. et al. Out of Himalaya: the impact of past Asian environmental changes on the evolutionary and biogeographical history of Dipodoidea (Rodentia). *J. Biogeogr.* **42**, 856-870 (2015).

5 Wu, S. Y. et al. The Evolution of Bipedalism in Jerboas (Rodentia: Dipodoidea): Origin in Humid and Forested Environments. *Evolution* **68**, 2108-2118 (2014).

6 Schenk, J. J., Rowe, K. C. & Steppan, S. J. Ecological Opportunity and Incumbency in the Diversification of Repeated Continental Colonizations by Muroid Rodents. *Syst. Biol.* **62**, 837-864 (2013).

7 Ding, L. Study on phylogeny of Cricetinae and evolution of *Cricetulus kamensis* (Rodentia, Cricetidae), Lanzhou University, (2020).

8 Neumann, K. et al. Molecular phylogeny of the Cricetinae subfamily based on the mitochondrial cytochrome b and 12S rRNA genes and the nuclear vWF gene. *Mol. Phylogenet. Evol.* **39**, 135-148 (2006).

9 Chevret, P. & Dobigny, G. Systematics and evolution of the subfamily Gerbillinae (Mammalia, Rodentia, Muridae). *Mol. Phylogenet. Evol.* **35**, 674-688 (2005).

10 Alhajeri, B. H. & Steppan, S. J. A phylogenetic test of adaptation to deserts and aridity in skull and dental morphology across rodents. *J. Mammal.* **99**, 1197-1216 (2018).

11. Fick, S. E. & Hijmans, R. J. WorldClim 2: new 1-km spatial resolution climate surfaces for global land areas. *Int. J. Climatol.* **37**, 4302-4315 (2017).

12. Pettorelli, N. et al. Using the satellite-derived NDVI to assess ecological responses to environmental change. *Trends. Ecol. Evol.* **20**, 503-510 (2005).

13. Phillips, S. J., Anderson, R. P. & Schapire, R. E. Maximum entropy modeling of species geographic distributions. *Ecol. Model.* **190**, 231-259 (2006).

14 Anderson, R. P. & Raza, A. The effect of the extent of the study region on GIS models of species geographic distributions and estimates of niche evolution: preliminary tests with montane rodents (genus Nephelomys) in Venezuela. *J. Biogeogr.* **37**, 1378-1393 (2010).

15. Elith, J. et al. A statistical explanation of MaxEnt for ecologists. *Divers. Distrib.* **17**, 43-57 (2011).

16. Phillips, S. J. & Dudík, M. Modeling of species distributions with Maxent: new extensions and a comprehensive evaluation. *Ecography* **31**, 161-175 (2008).

17. Liu, C., White, M. & Newell, G. Selecting thresholds for the prediction of species occurrence with presence-only data. *J. Biogeogr.* **40**, 778-789 (2013).

18. Vandermeer, J. H. Niche Theory. *Annu. Rev. Ecol. Syst.* **3**, 107-132 (1972).

19. Hurlbert, S. H. The Measurement of Niche Overlap and Some Relatives. *Ecology* **59**, 67-77 (1978).

20. Petraitis, P. S. Likelihood Measures of Niche Breadth and Overlap. *Ecology* **60**, 703-710 (1979).

21. Feinsinger, P., Spears, E. E. & Poole, R. W. A Simple Measure of Niche Breadth. *Ecology* **62**, 27-32 (1981).

22. Blonder, B., Lamanna, C., Violle, C. & Enquist, B. J. The n-dimensional hypervolume. *Global. Ecol. Biogeogr.* **23**, 595-609 (2014).

23. Broennimann, O. et al. Measuring ecological niche overlap from occurrence and spatial environmental data. *Global. Ecol. Biogeogr.* **21**, 481-497 (2012).

24. Schoener, T. W. Nonsynchronous spatial overlap of lizards in patchy habitats. *Ecology* **51**, 408-418 (1970).
