# Supplementary material for: Similar adaptative mechanism but divergent demographic history of four sympatric desert rodents in Eurasian inland
Source: Commun Biol. 2023 Jan 12;6:33. doi: 10.1038/s42003-023-04415-y (PMC9837166; doi:10.1038/s42003-023-04415-y)
Supplement: Supplementary file 2 — Description of Additional Supplementary Files [file 42003_2023_4415_MOESM2_ESM.docx]

**Description of Additional Supplementary Files**

**File name:** Supplementary Data 1

**Description:** Functional enrichments of the Biological Process GO terms and KEGG pathways of expanded genes in the four desert rodents

**File name:** Supplementary Data 2

**Description:** Functional enrichments of the Biological Process GO terms and KEGG pathways of contracted genes in the four desert rodents

**File name:** Supplementary Data 3

**Description:** Positively selected genes in *Dipus sagitta*

**File name:** Supplementary Data 4

**Description:** Positively selected genes in *Orientallactaga sibirica*

**File name:** Supplementary Data 5

**Description:** Positively selected genes in *Meriones meridianus*

**File name:** Supplementary Data 6

**Description:** Positively selected genes in *Phodopus roborovskii*

**File name:** Supplementary Data 7

**Description:** Rapidly evolving genes in *Dipus sagitta*

**File name:** Supplementary Data 8

**Description:** Rapidly evolving genes in *Orientallactaga sibirica*

**File name:** Supplementary Data 9

**Description:** Rapidly evolving genes in *Meriones meridianus*

**File name:** Supplementary Data 10

**Description:** Rapidly evolving genes in *Phodopus roborovskii*

**File name:** Supplementary Data 11

**Description:** Functional enrichments of the Biological Process GO terms of positively selected genes and rapidly evolved genes in the four desert rodents

**File name:** Supplementary Data 12

**Description:** Functional enrichments of the KEGG pathways of positively selected genes and rapidly evolved genes in the four desert rodents

**File name:** Supplementary Data 13

**Description:** Functional enrichments of KEGG pathways and top 100 Biological Process GO terms of Convergent genes in the four desert rodents

**File name:** Supplementary Data 14

**Description:** Functional enrichments associated with DNA damage repair of positively selected genes, rapidly evolved genes, and convergent genes in the four desert rodents

**File name:** Supplementary Data 15

**Description:** Source data of the Figures 1c, 2c, 2d, 5a, and 5b.
