# Supplementary material for: Similar adaptative mechanism but divergent demographic history of four sympatric desert rodents in Eurasian inland
Source: Commun Biol. 2023 Jan 12;6:33. doi: 10.1038/s42003-023-04415-y (PMC9837166; doi:10.1038/s42003-023-04415-y)
Supplement: Supplementary file 18 — reporting-summary [file 42003_2023_4415_MOESM18_ESM.pdf]

nature research

Corresponding author(s): Qisen Yang

Last updated by author(s): Dec 9, 2022

Reporting Summary

Nature Research wishes to improve the reproducibility of the work that we publish. This form provides structure for consistency and transparency in reporting. For further information on Nature Research policies, see our Editorial Policies and the Editorial Policy Checklist.

Statistics

For all statistical analyses, confirm that the following items are present in the figure legend, table legend, main text, or Methods section.

n/a	Confirmed	
☐	☒	The exact sample size (*n*) for each experimental group/condition, given as a discrete number and unit of measurement
☒	☐	A statement on whether measurements were taken from distinct samples or whether the same sample was measured repeatedly
☒	☐	The statistical test(s) used AND whether they are one- or two-sided<br>*Only common tests should be described solely by name; describe more complex techniques in the Methods section.*
☒	☐	A description of all covariates tested
☒	☐	A description of any assumptions or corrections, such as tests of normality and adjustment for multiple comparisons
☒	☐	A full description of the statistical parameters including central tendency (e.g. means) or other basic estimates (e.g. regression coefficient) AND variation (e.g. standard deviation) or associated estimates of uncertainty (e.g. confidence intervals)
☒	☐	For null hypothesis testing, the test statistic (e.g. *F*, *t*, *r*) with confidence intervals, effect sizes, degrees of freedom and *P* value noted<br>*Give P values as exact values whenever suitable.*
☒	☐	For Bayesian analysis, information on the choice of priors and Markov chain Monte Carlo settings
☒	☐	For hierarchical and complex designs, identification of the appropriate level for tests and full reporting of outcomes
☒	☐	Estimates of effect sizes (e.g. Cohen's *d*, Pearson's *r*), indicating how they were calculated

Our web collection on statistics for biologists contains articles on many of the points above.

Software and code

Policy information about availability of computer code

Data collection	no software used
Data analysis	guppy-v4-0-11, wtdbg2 v2.5, Racon, Quiver, Pilon, fragScaff, BWA v0.7.8, HiCUP v0.5.9, LACHESIS, ggplot2, BUSCO v3, LTR_FINDER v1.0.7, RepeatScout v1.0.5, RepeatModeler v1.0.3, RepeatMasker v4.0.6, RepeatProteinMask v4.0.7, TRF v4.07b, BLASTall v2.2.26, Solar v0.9.6, GeneWise v2.4.1, Trinity, IsoSeq3, TopHat2, Cufflinks, Augustus v2.7, Geneid v1.4, Genescan, GlimmerHMM v3.02, SNAP, GlimmerHMM, PASA2, EvidenceModeler v1.1.1, InterPro, MAXENT 3.4.0, ArcGIS Desktop 10.8.1, SAMtools, BCFtools, raster, dplyr, MUSCLE, Gblocks, RAxML v8.0.19, PAML 4.7, CAFÉ, KOBAS 3.0, Revigo.

For manuscripts utilizing custom algorithms or software that are central to the research but not yet described in published literature, software must be made available to editors and reviewers. We strongly encourage code deposition in a community repository (e.g. GitHub). See the Nature Research guidelines for submitting code & software for further information.

Data

Policy information about availability of data

All manuscripts must include a data availability statement. This statement should provide the following information, where applicable:

- Accession codes, unique identifiers, or web links for publicly available datasets
- A list of figures that have associated raw data
- A description of any restrictions on data availability

Genome assembly and the raw reads data of the four rodents have been deposited in NCBI under projection accession: PRJNA682294 (DS), PRJNA682295 (OS), PRJNA682296 (MM), and PRJNA682297 (PR).

Field-specific reporting

Please select the one below that is the best fit for your research. If you are not sure, read the appropriate sections before making your selection.

☐ Life sciences ☐ Behavioural & social sciences ☒ Ecological, evolutionary & environmental sciences

For a reference copy of the document with all sections, see nature.com/documents/nr-reporting-summary-flat.pdf

Ecological, evolutionary & environmental sciences study design

All studies must disclose on these points even when the disclosure is negative.

Study description	Phenotypes associated with metabolism and water retention are thought to be key to the adaptation of desert species. However, knowledge on the genetic changes and selective regimes on the similar and divergent ways to desert adaptation in sympatric and phylogenetically close desert organisms remains limited. Here, we generate a chromosome level genome assembly for Northern three-toed jerboa (Dipus sagitta) and three other high-quality genome assemblies for Siberian jerboa (Orientallactaga sibirica), Midday jird (Meriones meridianus), and Desert hamster (Phodopus roborovskii). Genomic analyses unveil that desert adaptation of the four species mainly result from similar metabolic pathways, such as arachidonic acid metabolism, thermogenesis, oxidative phosphorylation, insulin related pathway, DNA repair and protein synthesis and degradation. However, the specific evolved genes in the same adaptative molecular pathway often differ in the four species. We also reveal similar niche selection but different demographic histories and sensitivity to climate changes, which may be related to the diversified genomic adaptative features. In addition, our study suggests that nocturnal rodents have evolved some specific adaptative mechanism to desert environments compared to large desert animals. Our genomic resources will provide an important foundation for further research on desert genetic adaptations.
Research sample	Muscle samples were obtained from northern three-toed jerboa, Dipus sagitta, and Siberian jerboa, Orientallactaga sibirica, specimens collected from Tianshan, Inner Mongolia, China. Midday jird, Meriones meridianus, sample was collected from Dalaihubu, Inner Mongolia, China. Desert hamster, Phodopus roborovskii, sample was collected from Xilinhot, Inner Mongolia, China. All collected individuals were female.
Sampling strategy	Not Applicable
Data collection	All individuals were captured by rat traps or cages and sacrificed immediately with minimum suffering. Muscle tissues were collected and preserved in liquid nitrogen. All specimens and skulls collected in the field were deposited in the mammal collections of the National zoological Museum, IOZCAS. Jilong Cheng recorded.
Timing and spatial scale	Not Applicable
Data exclusions	Not Applicable
Reproducibility	Not Applicable
Randomization	Not Applicable
Blinding	Not Applicable

Did the study involve field work? ☒ Yes ☐ No

Field work, collection and transport

Field conditions	Tianshan: Climate cold winter, hot summer, spring breeze. The average annual precipitation is 360 mm, with a large annual variation and uneven distribution within the year. Most of them are concentrated in June to August, with northwest wind in winter and southwest wind in spring and autumn. The average annual wind speed is 3.5 m/s, the maximum wind speed can reach 21.7 m/s, and the number of strong wind days often reaches about 30 days.<br>Dalaihubu is a dry inland climate, with drought, less rain, evaporation, sufficient sunshine, large temperature difference, more sand and other climatic characteristics. The annual average temperature is 8.3, the average temperature in July is 26.6, the extreme high temperature is 42.5, the annual average average temperature is 8.6, the annual average precipitation is 37 mm, the annual extreme maximum precipitation is 103.0 mm, the annual minimum precipitation is 7.0 mm.<br>Xilinhot is a semi-arid continental climate in the middle temperate zone, with an average annual precipitation of 294.9 mm and a frost-free period of 110 days.
Location	Tianshan, Inner Mongolia, China.<br>Dalaihubu, Inner Mongolia, China.<br>Xilinhot, Inner Mongolia, China.
Access & import/export	Not Applicable
Disturbance	Not Applicable

Reporting for specific materials, systems and methods

We require information from authors about some types of materials, experimental systems and methods used in many studies. Here, indicate whether each material, system or method listed is relevant to your study. If you are not sure if a list item applies to your research, read the appropriate section before selecting a response.

Materials & experimental systems

n/a	Involved in the study
☒	☐ Antibodies
☒	☐ Eukaryotic cell lines
☒	☐ Palaeontology and archaeology
☐	☒ Animals and other organisms
☒	☐ Human research participants
☒	☐ Clinical data
☒	☐ Dual use research of concern

Methods

n/a	Involved in the study
☒	☐ ChIP-seq
☒	☐ Flow cytometry
☒	☐ MRI-based neuroimaging

Animals and other organisms

Policy information about studies involving animals; ARRIVE guidelines recommended for reporting animal research

Laboratory animals	Not Applicable
Wild animals	All individuals were captured by rat traps or cages and sacrificed immediately with minimum suffering. Muscle tissues were collected and preserved in liquid nitrogen. All specimens and skulls collected in the field were deposited in the mammal collections of the National zoological Museum, IOZCAS. Jilong Cheng recorded. Muscle samples were obtained from northern three-toed jerboa, Dipus sagitta, and Siberian jerboa, Orientallactaga sibirica, specimens collected from Tianshan, Inner Mongolia, China. Midday jird, Meriones meridianus, sample was collected from Dalaihubu, Inner Mongolia, China. Desert hamster, Phodopus roborovskii, sample was collected from Xilinhot, Inner Mongolia, China. All collected individuals were female.
Field-collected samples	Not Applicable
Ethics oversight	All procedures were conducted in accordance with Animal Research Protocol IOZ-2006 approved by the Animal Care Committee of the Institute of Zoology, Chinese Academy of Sciences (IOZCAS).

Note that full information on the approval of the study protocol must also be provided in the manuscript.
